# Prevalence and prognostic relevance of perioperative myocardial injury/infarction after major noncardiac surgery in older patients

**DOI:** 10.1093/ageing/afag103

**Published:** 2026-04-20

**Authors:** Emre Ergin, Koray Durak, Noemi Glarner, Fisnik Haziri, Katrin Burri-Winkler, Emel Kaplan, Gabrielle Huré, Vanessa Thommen, Mirjam Pargger, Edoardo Usai, Daniel Bolliger, Luzius Steiner, Edin Mujagic, Didier Lardinois, Stefan Schaeren, Andreas Mueller, Franz Haller, Nikolaus Buchmann, Heike A Bischoff-Ferrari, Felix Mahfoud, Ivo Strebel, Danielle M Gualandro, Christian Puelacher, Christian Mueller

**Affiliations:** Department of Cardiology, University Hospital Basel, Basel, Basel-Stadt, Switzerland; Cardiovascular Research Institute Basel, University Hospital Basel, Basel, Basel-Stadt, Switzerland; Department of Cardiology, University Hospital Basel, Basel, Basel-Stadt, Switzerland; Cardiovascular Research Institute Basel, University Hospital Basel, Basel, Basel-Stadt, Switzerland; Department of Cardiology, University Hospital Basel, Basel, Basel-Stadt, Switzerland; Cardiovascular Research Institute Basel, University Hospital Basel, Basel, Basel-Stadt, Switzerland; Department of Cardiology, University Hospital Basel, Basel, Basel-Stadt, Switzerland; Cardiovascular Research Institute Basel, University Hospital Basel, Basel, Basel-Stadt, Switzerland; Department of Cardiology, University Hospital Basel, Basel, Basel-Stadt, Switzerland; Cardiovascular Research Institute Basel, University Hospital Basel, Basel, Basel-Stadt, Switzerland; Anesthesiology, University Hospital Basel, Basel, Basel-Stadt, Switzerland; Department of Cardiology, University Hospital Basel, Basel, Basel-Stadt, Switzerland; Cardiovascular Research Institute Basel, University Hospital Basel, Basel, Basel-Stadt, Switzerland; Department of Cardiology, University Hospital Basel, Basel, Basel-Stadt, Switzerland; Cardiovascular Research Institute Basel, University Hospital Basel, Basel, Basel-Stadt, Switzerland; Department of Cardiology, University Hospital Basel, Basel, Basel-Stadt, Switzerland; Cardiovascular Research Institute Basel, University Hospital Basel, Basel, Basel-Stadt, Switzerland; Department of Cardiology, University Hospital Basel, Basel, Basel-Stadt, Switzerland; Cardiovascular Research Institute Basel, University Hospital Basel, Basel, Basel-Stadt, Switzerland; Department of Cardiology, University Hospital Basel, Basel, Basel-Stadt, Switzerland; Cardiovascular Research Institute Basel, University Hospital Basel, Basel, Basel-Stadt, Switzerland; Anesthesiology, University Hospital Basel, Basel, Basel-Stadt, Switzerland; Anesthesiology, University Hospital Basel, Basel, Basel-Stadt, Switzerland; Department of Clinical Research, University of Basel, Basel, Basel-Stadt, Switzerland; Department of Vascular Surgery, University Hospital Basel, Basel, Basel-Stadt, Switzerland; Department of Thoracic Surgery, University Hospital Basel, Basel, Basel-Stadt, Switzerland; Department of Spinal Surgery, University Hospital Basel, Basel, Basel-Stadt, Switzerland; Department of Orthopaedics and Trauma Surgery, University Hospital Basel, Basel, Basel-Stadt, Switzerland; Department of Geriatric Medicine Felix Platter, University of Basel, Basel, Basel-Stadt, Switzerland; Department of Geriatric Medicine Felix Platter, University of Basel, Basel, Basel-Stadt, Switzerland; Department of Geriatric Medicine Felix Platter, University of Basel, Basel, Basel-Stadt, Switzerland; Department of Cardiology, University Hospital Basel, Basel, Basel-Stadt, Switzerland; Cardiovascular Research Institute Basel, University Hospital Basel, Basel, Basel-Stadt, Switzerland; Department of Cardiology, University Hospital Basel, Basel, Basel-Stadt, Switzerland; Cardiovascular Research Institute Basel, University Hospital Basel, Basel, Basel-Stadt, Switzerland; Department of Cardiology, University Hospital Basel, Basel, Basel-Stadt, Switzerland; Cardiovascular Research Institute Basel, University Hospital Basel, Basel, Basel-Stadt, Switzerland; Department of Cardiology, University Hospital Basel, Basel, Basel-Stadt, Switzerland; Cardiovascular Research Institute Basel, University Hospital Basel, Basel, Basel-Stadt, Switzerland; Department of Internal Medicine III, Cardiology and Angiology, Medical University of Innsbruck, Innsbruck, Tyrol, Austria; Department of Cardiology, University Hospital Basel, Basel, Basel-Stadt, Switzerland; Cardiovascular Research Institute Basel, University Hospital Basel, Basel, Basel-Stadt, Switzerland

**Keywords:** perioperative, ageing, myocardial injury, cardiovascular, older people

## Abstract

**Background:**

The prognostic relevance of perioperative myocardial injury/infarction (PMI) in older patients undergoing major noncardiac surgery remains unclear, as high comorbidity burden may lessen its impact.

**Methods:**

Older patients (defined as age ≥70 years with ≥3 comorbidities, or ≥80 years) enrolled in a multicentre, prospective study of patients at increased cardiovascular risk undergoing major noncardiac surgery were analysed. The primary endpoint, all-cause mortality at 1 year, was analysed using Cox proportional hazards regression. Secondary endpoints included major adverse cardiac events (MACE) (cardiovascular death, acute myocardial infarction, life-threatening arrhythmia and acute heart failure), analysed using Fine–Grey hazard regression. All models were adjusted for prespecified confounders with PMI as a time-varying exposure.

**Results:**

Amongst 4634 older patients (median age 80 years; 42.9% women), PMI occurred in 892 patients (19.2%), which was higher than in younger patients (*P* < .0001). The distribution of PMI aetiologies was comparable between groups. At 1 year, all-cause mortality was 26.2% in patients with PMI and 13.2% in patients without PMI, and MACE occurred in 30% versus 13%, respectively. After multivariable adjustment, the hazard ratio of PMI was highest on postoperative day 1 (all-cause mortality: 10.5 [95% CI 4.5–24.5]; MACE: 4.4 [95% CI 3.2–5.9]), declined by day 90 (1.4 [95% CI 1.0–1.9] and 2.2 [95% CI 1.7–2.7], respectively), and persisted through 1 year.

**Conclusions:**

PMI was very common amongst older patients and associated with substantially higher 1-year risks of all-cause mortality and MACE, with greatest vulnerability observed during the initial 90 postoperative days.

## Key points

- Perioperative myocardial injury/infarction (PMI) is common in older patients undergoing major noncardiac surgery.
- Nearly one in five older patients developed PMI, a higher rate than in younger patients.
- PMI was associated with substantially increased risks of 1-year all-cause mortality and major adverse cardiac events.
- The excess risk was greatest in the early postoperative period and remained elevated throughout the first year.

## Introduction

Each year, ~300 million surgeries are performed with a steadily increasing number involving older patients [1]. Perioperative myocardial injury/infarction (PMI) is defined as a transient, often mild and asymptomatic increase in cardiac troponin (cTn) during major noncardiac surgery [2–4]. Most older patients are at an increased cardiovascular risk, and recent observations suggest that PMI is more common than previously recognised, raising important clinical concerns [2, 5–8].

However, the prevalence and prognostic relevance of PMI in older patients remain incompletely understood. On the one hand, modelling studies suggested that in the overall surgical high-risk population PMI has the highest population attributable risk for death amongst postoperative complications [8, 9]. Accordingly, some clinical practice guidelines recommend active surveillance for PMI in patients at high risk [2, 4, 6, 7]. On the other hand, older patients often have multiple severe chronic comorbidities and the development of PMI may be considered more a reflection of severe comorbidities rather than having independent prognostic relevance [10, 11]. Accordingly, other clinical practice guidelines do not recommend active surveillance for PMI [12, 13].

To address this major uncertainty, we performed a large prospective multicentre study to assess the prevalence and prognostic relevance of PMI for all-cause mortality and major adverse cardiac events (MACE) during long-term follow-up in consecutive older patients undergoing major noncardiac surgery.

## Methods

### Study design

This was a secondary analysis within a prospective, multicentre cohort [4, 8, 12, 14–17]. Patients provided written general consent to registration in a dedicated prospective database, and the study was approved by the local ethics committees. We adhered to the recommendations of the Strengthening the Reporting of Observational Studies in Epidemiology (STROBE) statement (Appendix 1) [18] and the study was conducted in accordance with the Declaration of Helsinki [19].

### Population

We included consecutive patients at increased cardiovascular risk undergoing major inpatient noncardiac surgery at a university hospital and a cantonal hospital in Switzerland, and a university hospital in Brazil, who were eligible for the institutional active PMI surveillance (Appendix 2**—**Population). For our analysis, we included older patients with at least two cTn measurements (high-sensitivity cTnT [hs-cTnT] in the university hospitals in Switzerland and Brazil; sensitive cTnI [s-cTnI] in the cantonal hospital in Switzerland). Older patients were defined as either ≥70 years of age with multimorbidity or ≥80 years of age regardless of multimorbidity according to local standards of the Swiss Frailty Network and Repository and other European societies [20–26] (further details in Appendix 2—Population). Multimorbidity was defined as at least three comorbidities from the cardiovascular assessment of patients undergoing noncardiac surgery according to the European Society of Cardiology [2]. These were chronic heart failure (CHF), hypertension, diabetes mellitus, PAD, previous stroke or transient ischaemic attack, CAD, chronic obstructive pulmonary disease (COPD) or restrictive lung disease (hereafter referred to as chronic lung disease), active cancer and chronic kidney disease (CKD). Reduced functional capacity was routinely reported during preoperative anaesthesiology assessment (PAS) and was defined as <4 metabolic equivalent tasks (METs) [27]. We excluded patients who did not meet active surveillance criteria (<45 years, <24 h hospital stay, surgery involving the heart; Appendix 2**—**Population), had their surgery cancelled, had cardiac surgery or spontaneous MI within 14 days before surgery, and patients with incomplete PMI documentation/work-up not allowing the accurate central adjudication of the PMI aetiology. Furthermore, we excluded (1) patients undergoing palliative surgery and (2) patients who were already included in the study within the previous 12 months to analyse 1-year endpoints.

### Definition of PMI

PMI was defined as an absolute increase in the (h)s-cTn concentration of at least the upper limit of normal (ULN) for each assay above the preoperative concentration (≥14 ng/L for hs-cTnT [Elecsys Gen 5] and ≥ 45 ng/L for s-cTnI [Vista]), or between two postoperative concentrations (if the preoperative measurement was missing) within 3 days after surgery. PMI diagnosis was made based on the (h)s-cTn criterion only and did not require symptoms or electrocardiogram (ECG) changes. PMI aetiology was centrally adjudicated by two independent experts based on all available clinical data obtained during index hospitalisation (Appendix 2**—**PMI definition).

### Follow-up and prognostic endpoints

Patients and their primary care physicians were contacted at 365 days to assess all-cause mortality (primary prognostic endpoint) and MACE. MACE was defined as a composite endpoint comprising cardiovascular death, acute myocardial infarction (AMI), life-threatening arrhythmia and acute heart failure (AHF). The definition of life-threatening arrhythmia included ventricular fibrillation, sustained ventricular tachycardia and third-degree atrioventricular block. Death was classified as non-cardiovascular or cardiovascular in accordance with recent guidelines [28].

### Exploratory survey

Additionally, board-certified geriatricians at a university geriatric hospital in Switzerland were asked to participate in a survey assessing their expert opinion regarding the prognostic relevance of PMI (Appendix 3). Participants were informed about the topic, including the definition of PMI, and then asked if they assumed that PMI would have a significant independent impact on (a) 1-year all-cause mortality and (b) 1-year MACE including acute heart failure, life-threatening arrhythmia, myocardial infarction and cardiovascular death in older patients.

### Statistical analysis

Continuous variables were shown as medians and interquartile ranges, categorical variables as numbers and percentages, and compared using either Chi-square or Fisher’s exact test. Baseline characteristics were stratified by exposure group. One-year all-cause mortality was plotted using cumulative incidences for patients with PMI and patients without PMI, respectively. For 1-year MACE events, the cumulative incidence function method was used for plotting the incidence of the occurrence of the composite outcome whilst taking competing risk (non-cardiovascular death) into account with Grey’s test for comparison between groups.

Sample size calculations for regression modelling were performed a priori to ensure adequate precision and stability of effect estimates, and to guide the prespecified number of degrees of freedom (Appendix 2—Sample size calculation). Confounders of all-cause mortality and MACE were prespecified in a directed acyclic graph (DAG; Appendix 4) according to previous literature [13, 27, 29, 30]. The prespecified confounders included age, ESC surgical risk (low, medium, high surgery-related risk for perioperative cardiac events) [2], active cancer, history of CHF, PAD, CAD, CKD, diabetes mellitus, history of stroke/TIA, hypertension, reduced functional capacity, chronic lung disease, preoperative haemoglobin, centre of surgery and surgical discipline. Sex was not adjusted for, as we expect little difference in cardiovascular risk between the sexes in the older population [29]. For all-cause mortality, a Cox proportional hazards regression was used to estimate cause-specific hazard ratios of PMI. The proportionality assumption was relaxed by incorporating a Royston–Parmar model. For MACE, a Fine–Grey hazard regression was chosen to account for non-cardiovascular death as a competing event and estimate subdistribution hazard ratios of PMI to prevent censoring of patients after death and the assumption that they remained event-free for the entire follow-up. PMI was entered as a time-varying exposure, and non-linear relationships were adjusted with restricted cubic splines.

All statistical comparisons were two-sided, and *P*-values of <.05 indicated statistically significant differences. All statistical analyses were performed using R 4.3.3 (R Foundation for Statistical Computing, Vienna, Austria) and packages (with version numbers) are listed in Appendix 5.

### Sensitivity analysis

To address potentially different outcomes in patients who survived the early postoperative period and are eligible for geriatric rehabilitation programmes, we performed a sensitivity analysis excluding patients who died before hospital discharge. Additionally, a second sensitivity analysis with geriatric profile patients (defined as having ≥3 comorbidities and reported reduced functional capacity [<4 METs]) independent of age was performed. Thirdly, we performed sensitivity analyses with different age cut-offs defining older patients at 60, 70 and 80 years of age regardless of comorbidities.

## Results

### Baseline characteristics

Amongst 16 204 patients enrolled between September 2012 and September 2019, 4634 patients were eligible for this analysis (Appendix 6). The median age was 80 years, 42.9% of patients were female, and 67.8% fulfilled the criteria of multimorbidity. The most frequent comorbidities were hypertension (81.6%), CKD (45.8%) and CAD (41.1%), and 47.3% had reduced functional capacity (Table 1).

**Table 1:** Baseline characteristics.

Baseline	Overall	No PMI	PMI
n (%)	4634	3742 (80.8)	892 (19.2)
Age (median [IQR])	80.0 [75.0, 83.0]	80.0 [75.0, 83.0]	80.0 [76.0, 83.0]
Female gender, n (%)	1989 (42.9)	1635 (43.7)	354 (39.7)
Multimorbidity, n (%)	3144 (67.8)	2491 (66.6)	653 (73.2)
Polypharmacy, n (%)	2160 (46.6)	1708 (45.6)	452 (50.7)
Reduced functional capacity, n (%)	2192 (47.3)	1697 (45.4)	495 (55.5)
**Comorbidities**			
Chronic heart failure, n (%)	1080 (23.3)	809 (21.6)	271 (30.4)
Diabetes mellitus, n (%)	1483 (32.0)	1191 (31.8)	292 (32.7)
Peripheral artery disease, n (%)	1552 (33.5)	1202 (32.1)	350 (39.2)
Previous stroke or TIA, n (%)	788 (17.0)	626 (16.7)	162 (18.2)
Hypertension, n (%)	3706 (81.6)	2974 (81.0)	732 (84.3)
Chronic kidney disease, n (%)	2080 (45.8)	1605 (43.7)	475 (54.7)
Active cancer, n (%)	640 (14.1)	543 (14.8)	97 (11.2)
COPD, n (%)	872 (19.2)	701 (19.1)	171 (19.7)
Chronic restrictive lung disease, n (%)	108 (2.6)	84 (2.5)	24 (3.2)
Coronary artery disease, n (%)	1903 (41.1)	1471 (39.3)	432 (48.4)
**Surgery details**			
Surgical Discipline, n (%)			
Ortho/Trauma	1457 (31.4)	1155 (30.9)	302 (33.9)
Spinal	590 (12.7)	471 (12.6)	119 (13.3)
Thoracic	263 (5.7)	196 (5.2)	67 (7.5)
Visceral	603 (13.0)	516 (13.8)	87 (9.8)
Urology	468 (10.1)	415 (11.1)	53 (5.9)
Neurosurgery	13 (0.3)	11 (0.3)	2 (0.2)
Vascular	1194 (25.8)	939 (25.1)	255 (28.6)
Other	46 (1.0)	39 (1.0)	7 (0.8)
ESC surgery risk, n (%)			
ESC <1%	963 (21.2)	846 (23.0)	117 (13.5)
ESC 1%–5%	2936 (64.7)	2345 (63.8)	591 (68.1)
ESC >5%	642 (14.1)	482 (13.1)	160 (18.4)
Urgency of surgery, n (%)			
Urgent within 24 h	539 (11.9)	406 (11.1)	133 (15.3)
Urgent after 24 h	937 (20.6)	731 (19.9)	206 (23.7)
Elective	3065 (67.5)	2536 (69.0)	529 (60.9)

Baseline characteristics shown in number and percentage of overall cohort, stratified by No PMI- and PMI-groups. Age is shown as median with interquartile ranges (IQR). Abbreviations: TIA—transient ischaemic attack, COPD—chronic obstructive pulmonary disease, ESC—European Society of Cardiology.

### PMI incidence, patient characteristics and aetiologies

PMI occurred in 892 patients (19.2%). Patients developing PMI had higher rates of CAD (48.4% vs 39.3%), CHF (30.4% vs 21.6%) and CKD (54.7% vs 43.7%) versus those who did not. The aetiology of PMI was centrally adjudicated as primarily extracardiac in 85 patients (9.5%), type 1 myocardial infarction (T1MI) in 66 (7.4%), tachyarrhythmia in 45 (5%), AHF in 58 (6.5%) and likely type 2 myocardial infarction (T2MI) in 638 (71.5%; Table 2). The incidence of PMI was significantly higher in older patients than in younger patients (19.2% vs. 10.7%, *P* < .0001), with a similar distribution (Appendix 7).

**Table 2:** Endpoints following different PMI aetiologies.

Endpoints	Overall PMI	Extracardiac	T1MI	Tachyarrhythmia	AHF	Likely T2MI
n (%)	892	85 (9.5)	66 (7.4)	45 (5)	58 (6.5)	638 (71.5)
**All-cause death, n (%)**	**230 (26.2)**	**46 (54.1)**	**22 (33.8)**	**19 (43.2)**	**26 (44.8)**	**117 (18.7)**
Cardiovascular death, n (%)	129 (14.5)	19 (22.4)	20 (30.3)	11 (24.4)	17 (29.3)	62 (9.7)
Non-cardiovascular death, n (%)	101 (11.3)	27 (31.8)	2 (3.0)	8 (17.8)	9 (15.5)	55 (8.6)
**MACE without CV-death, n (%)**	**187 (21.0)**	**21 (24.7)**	**31 (48.4)**	**16 (35.6)**	**25 (43.1)**	**94 (14.8)**
Acute myocardial infarction, n (%)	49 (5.5)	3 (3.5)	16 (24.2)	2 (4.4)	4 (6.9)	24 (3.8)
Acute heart failure, n (%)	132 (14.8)	12 (14.1)	23 (34.8)	12 (26.7)	19 (32.8)	66 (10.3)
Life-threatening arrhythmia, n (%)	36 (4.0)	9 (10.6)	4 (6.1)	4 (8.9)	5 (8.6)	14 (2.2)

Number and percentage of all-cause death and MACE at 1 year in older patients with PMI according to different PMI aetiologies. MACE defined as composite endpoint; therefore, total number of individual events exceeds total number of composite endpoints. Abbreviations: MACE—major adverse cardiac events, CV-death—cardiovascular death, T1MI—Type 1 myocardial infarction, AHF—acute heart failure, likely T2MI—likely type 2 myocardial infarction. Values shown in bold represent composite categories, while the subsequent non-bold entries denote the individual components comprising each category.

### Primary prognostic endpoint: all-cause mortality

**Figure 1:**
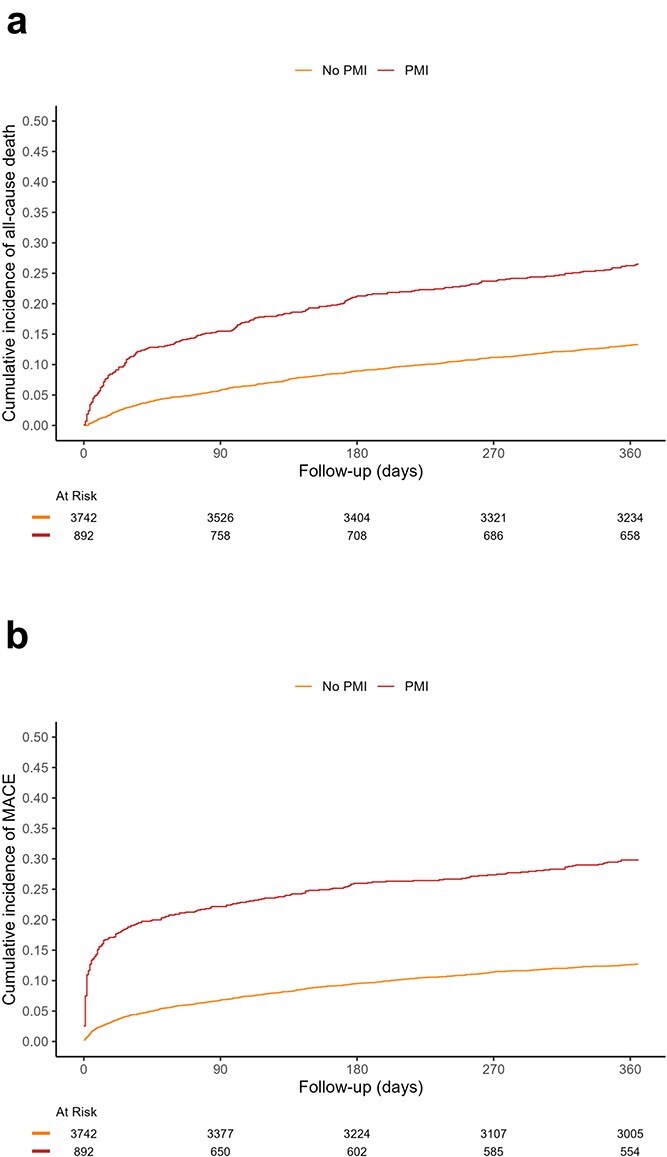
(a) Cumulative incidence of all-cause mortality and (b) Cumulative incidence of MACE: 101 patients were omitted from analysis due to missing follow-up data. Below the x-axes the number of at-risk patients are shown at corresponding timepoints on the respective x-axis.

**Figure 2:**
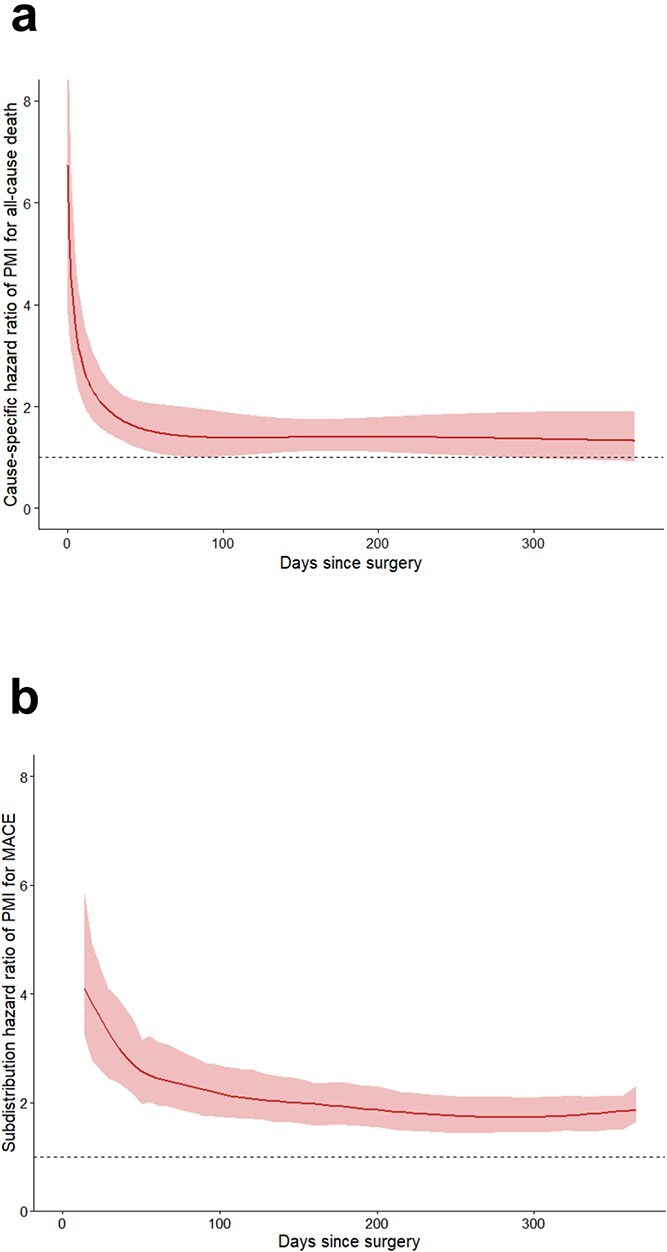
(a) Cause-specific hazard ratio of PMI for all-cause mortality and (b) Subdistribution hazard ratio of PMI for MACE: All-cause mortality (part A) and MACE (part B) within 1 year stratified according to PMI after non-cardiac surgery, including adjusted cause-specific hazard ratios (part A) and adjusted subdistribution hazard ratios (part B) from multivariable hazard analysis (adjusted for age, preoperative haemoglobin, surgical discipline, centre of surgery, ESC surgery risk [low, medium, high risk of cardiac events], heart failure, coronary artery disease, active cancer, chronic kidney disease, chronic lung disease [COPD, chronic restrictive lung disease], diabetes mellitus, history of stroke/transient ischaemic attack, hypertension, peripheral artery disease, reduced functional capacity). Abbreviations: MACE—Major adverse cardiac events, PMI—Perioperative myocardial infarction/injury, COPD—Chronic obstructive pulmonary disease.

One-year follow-up was complete in 4533/4634 patients (97.8%). Patients developing PMI had nearly double the all-cause mortality within 1 year versus patients without PMI (26.2% vs 13.2%; Table 3) and cumulative incidences were significantly higher **(**log-rank *P* < .001; Figure 1a). In patients with PMI, cardiovascular death was the leading cause of death, whereas non-cardiovascular death was the leading cause of death in patients without PMI. After multivariable adjustment, the cause-specific hazard ratio of PMI for all-cause mortality was 10.5 (95% CI 4.5–24.5) on the first postoperative day, which decreased to 1.4 (95% CI 1.0–1.9) after 90 days (Appendix 8) and then remained stable through 1 year after surgery (Figure 2a). Reduced functional capacity was a strong confounder with an adjusted hazard ratio of 1.9 (95% CI 1.6–2.3) for all-cause mortality. In contrast, chronological age was not associated with all-cause mortality (Appendix 9).

**Table 3:** Endpoints.

Endpoints	Overall	No PMI	PMI
n	4634	3742	892
**All-cause death, n (%)**	**717 (15.7)**	**487 (13.2)**	**230 (26.2)**
Cardiovascular death, n (%)	327 (7.1)	198 (5.3)	129 (14.5)
Non-cardiovascular death, n (%)	390 (8.4)	289 (7.7)	101 (11.3)
**MACE without CV-death, n (%)**	**519 (11.2)**	**332 (8.9)**	**187 (21.0)**
Acute myocardial infarction, n (%)	133 (2.9)	84 (2.2)	49 (5.5)
Acute heart failure, n (%)	386 (8.3)	254 (6.8)	132 (14.8)
Life-threatening arrhythmia, n (%)	73 (1.6)	37 (1.0)	36 (4.0)

Number and percentage of all-cause death and MACE at 1 year in the overall cohort after accounting for cases which are lost to follow-up, stratified by No PMI- and PMI-group. MACE defined as composite endpoint; therefore, total number of individual events exceeds total number of composite endpoints. Abbreviations: MACE—major adverse cardiac events, CV-death—cardiovascular death. Values shown in bold represent composite categories, while the subsequent non-bold entries denote the individual components comprising each category.

### Secondary endpoint: MACE

Patients with PMI had more than twice the incidence of MACE within 1 year compared to patients without PMI (30% vs 13%; Grey’s test *P* < .001; Figure 1b). This excess risk was consistent for all components, including AMI (5.5% vs 2.2%), AHF (14.8% vs 6.8%) and life-threatening arrhythmia (4.0% vs 1.0%). After multivariable adjustment, the subdistribution hazard ratio of PMI for MACE was 4.4 (95% CI 3.2–5.9) on postoperative day 1, decreased to 2.2 (95% CI 1.7–2.7) on postoperative day 90 (Appendix 10), and remained constant until 1 year after surgery (Figure 2b). Reduced functional capacity was a strong confounder with an adjusted hazard ratio of 1.7 (95% CI 1.3–2.1) for MACE. In contrast, age did not show a relevant association with MACE (Appendix 11).

### Sensitivity analysis

A prespecified sensitivity analysis with patients who survived until hospital discharge (n = 4326 for all-cause mortality and 4476 for MACE) showed similar results regarding all-cause mortality and MACE within 1 year (Appendix 12). The second and third sensitivity analyses (with geriatric profile patients independent of age and age cut-offs at 60, 70 and 80 years regardless of comorbidities) also showed similar results regarding all-cause mortality and MACE within 1 year (Appendices 13 and 14).

### Exploratory survey

In the exploratory survey, 6 of 14 (43%) board-certified geriatricians did not consider PMI to be an independent predictor of all-cause mortality at 1 year (Appendix 15).

## Discussion

This analysis of a large, prospective, multicentre cohort addressed major uncertainties regarding the independent prognostic relevance of PMI in older patients. We report seven major findings:

First, in the prospective study nearly one in five older patients (19.2%) developed PMI, resulting in a higher incidence compared to the overall population [4, 8, 12, 14–17]. Second, the distribution of PMI aetiologies in older patients was comparable to younger patients, and included T1MI in 7.4%, tachyarrhythmia in 5%, AHF in 6.5% and likely T2MI in 71.5%. Third, older patients with PMI had nearly double the all-cause mortality and more than double the incidence of MACE at 1 year compared to patients without PMI. The excess risk associated with PMI was most pronounced within the first 90 days after surgery and remained elevated, albeit to a lesser degree, through the first year. Fourth, the associations of PMI with both all-cause mortality and MACE persisted after multivariable adjustment, indicating that the high event rate observed is not just a reflection of comorbidities. Fifth, reduced functional capacity—closely linked to frailty [31, 32]—but not chronological age, was a major confounder for all-cause mortality and MACE. Sixth, results were consistent in sensitivity analyses restricted to patients discharged alive, underscoring the relevance of these findings for clinicians and geriatricians in rehabilitation and post-acute care settings. Seventh, in the exploratory survey nearly half of board-certified geriatricians did not consider PMI to be an independent predictor of all-cause mortality after major noncardiac surgery in older patients.

These findings extend and corroborate prior studies that had primarily focused on perioperative, in-hospital or 30-day postoperative MACE amongst older adults, consistently demonstrating a greater risk compared to younger patients [9, 33–36]. In a retrospective single-centre cohort study of 2984 patients aged ≥80 years who underwent major noncardiac surgery from 2011 to 2021 and had a conventional cTnT assay measurement within the first 3 days after surgery, the incidence of PMI was 14% and patients with PMI had higher 30-day and 1-year mortality compared to patients without PMI. The lack of baseline cTn measurements, the use of less sensitive cTnT methods to detect PMI, differences in definition of PMI and patient characteristics, and different durations of follow-up need to be considered when directly comparing the two studies. Our finding regarding the importance of reduced functional capacity and its association with frailty is further supported by a meta-analysis of older adults undergoing elective surgery, which identified established geriatric syndromes including functional impairment, frailty and cognitive impairment, being associated with postoperative complications, whereas chronological age or the American Society of Anesthesiologists (ASA) score was not [37].

The observed time-dependency of the extent of the excess risk associated with PMI has important implications for cardiovascular management of older patients with PMI who may potentially benefit from the following established therapies: First, optimisation of CHF medication that has been shown to be effective in older patients [38–40]. Second, a high proportion of patients with PMI are not receiving guideline-recommended cholesterol-lowering therapies [41]. Statin therapy has demonstrated efficacy as a secondary or tertiary prevention strategy for MACE also in older patients [42]. Of note, in adults aged 80 years with polypharmacy and/or frailty, the effectiveness remains less certain due to limited evidence and its prescription should be based on individualised decision making [43]. Third, percutaneous coronary interventions (PCI) should be considered in patients with PMI due to T1MI. However, uncertainties remain regarding the beneficial effect of coronary revascularisation in older patients with NSTEMI [44, 45]. Fourth, postoperative cardiology consultations were associated with improved long-term outcomes in patients with PMI, underscoring the need to complement active surveillance for PMI with cardiology evaluation in those developing PMI [46, 47]. Future studies are warranted to investigate these strategies for older patients in greater depth.

Our findings are of potential relevance for future research and geriatric rehabilitation centres as they play central roles in the postoperative care of older patients. As nearly half of board-certified geriatricians in our exploratory survey considered PMI not to be associated with an increased risk of all-cause mortality at 1 year, improving physician awareness of the elevated risk in patients with PMI and further investigations for optimal treatment strategies are of relevance. A heightened awareness and a proactive attitude towards optimisation of cardiovascular therapy during geriatric rehabilitation could potentially benefit older patients with PMI, as many MACE occurred during the typical rehabilitation window [48]. Furthermore, reduced functional capacity was significantly associated with PMI and contributes to the need of assistance with activities of daily living (ADL) and increased geriatric rehabilitation utilisation. On the other hand, multidomain rehabilitative measures have been shown to help regain baseline functional capacity [49], and may be particularly effective in older patients [50]. In a recent multicentre, randomised trial involving 512 patients, median age 80 years, with impaired physical performance 1 month after AMI, an intervention consisting of control of cardiovascular risk factors, dietary counselling and exercise training reduced the composite of cardiovascular death or unplanned hospitalisation for cardiovascular causes within 1 year [50].

### Strengths

Our study has several strengths that reinforce the validity and clinical relevance of our findings. The analysis was conducted in a large, multicentre, prospective cohort with long-term follow-up for clinically relevant endpoints and had a very high rate of complete follow-up (>97%). Sample size for model derivation was determined to be sufficient by a prespecified sample size calculation including relevant covariates. PMI was centrally adjudicated by two independent clinicians with classification of the underlying aetiology. Hazard ratios of PMI were modelled with a time-varying effect allowing for better interpretation of long-term outcomes. Additionally, we reported subdistribution hazard ratios for MACE which provide a more realistic estimate addressing the competing risk of non-cardiovascular death.

### Limitations

The following limitations warrant consideration. First, there is no universally accepted definition of PMI. However, we adhered to the definition recommended by the European Society of Cardiology [2]. Second, this cohort included patients undergoing major noncardiac surgery. The rate of MACE and mortality associated with PMI occurring in patients undergoing minor or outpatient surgery remain unknown. Third, a classification of frailty for subgroup analysis and confounder adjustment was not possible, as we did not perform structured questionnaires or clinical assessment of frailty at enrolment. Nevertheless, we included a measurement of reduced functional capacity which was systematically obtained at preoperative anaesthesiology assessments and is closely linked to frailty [31, 32]. Fourth, as an observational study, causality between PMI and the investigated endpoints cannot be established, and the findings should be interpreted as associations. Randomised controlled trials are necessary to investigate treatment options and their impact on long-term outcomes in this population. Fifth, the conducted exploratory survey amongst board-certified geriatricians does not adequately represent the specialty due to its small sample size.

## Conclusions

In this multicentre, older cohort, PMI was associated with substantially and incrementally increased risks of all-cause mortality and MACE, with the greatest vulnerability in the first 90 days after surgery. Whilst the excess mortality risk attenuated over time, the long-term risk of MACE, particularly AHF, remained elevated. These findings highlight the importance of increased clinical awareness for PMI, active surveillance and geriatric rehabilitation strategies. Further research is necessary to investigate the optimisation of cardiac risk factors in this population.

## Supplementary Material

Appendix_1_afag103

Appendix_2_afag103

Appendix_3_afag103

Appendix_4_afag103

Appendix_5_afag103

Appendix_6_afag103

Appendix_7_afag103

Appendix_8_afag103

Appendix_9_afag103

Appendix_10_afag103

Appendix_11_afag103

Appendix_12_afag103

Appendix_13_afag103

Appendix_14_afag103

Appendix_15_afag103

aa-25-3712-File002_afag103
