# Supplementary material for: Prevalence and prognostic relevance of perioperative myocardial injury/infarction after major noncardiac surgery in older patients
Source: Age Ageing. 2026 Apr 20;55(4):afag103. doi: 10.1093/ageing/afag103 (PMC13092811; doi:10.1093/ageing/afag103)
Supplement: Appendix_2_afag103 [file appendix_2_afag103.docx]

**Appendix 2: Supplementary Methods**

**Population**

Patients were eligible for the institutional active PMI surveillance and included if they were considered at increased cardiovascular risk, defined as ≥65 years of age or ≥45 years with a history of coronary artery disease (CAD), peripheral artery disease (PAD), or stroke/transient ischaemic attack (TIA), undergoing major inpatient noncardiac surgery with a planned postoperative stay of ≥24 h [2–8].

The definition of older patients was established according to 1) the Swiss Frailty Network and Repository, other European societies and local standards [9–14], and 2) based on meta-analyses showing increased prevalence of frailty and research showing an increase in morbidity and mortality burden at ≥70 years of age [15–18].

**PMI definition**

PMI aetiology was centrally adjudicated by two independent experts based on all available clinical data, including ECG, serial laboratory measurements including cTn and haemoglobin, vital sign monitoring in the peri- and intraoperative period, and echocardiography, cardiac stress testing, and coronary angiography if performed. In cases of disagreement between the two adjudicating reviewers, consensus was sought and found by discussion with a third reviewer. PMI was hierarchically classified based on the likely trigger for myocardial injury or infarction, aiming to reflect different clinical management pathways [7,8,19,20]. Following classification was applied: (1) extracardiac if caused by a primarily extracardiac disease such as severe sepsis, stroke, pulmonary embolism, or blunt or surgical cardiac trauma; (2) cardiac, further subtyped into type 1 myocardial infarction (T1MI), tachyarrhythmia, or AHF; (3) cardiac, likely type 2 myocardial infarction (LT2MI) if the causes mentioned above (1, 2) could be ruled out and additionally a documented or suspected type 2 trigger (e.g. severe hypotension, anaemia, hypoxia, sinus tachycardia) was present [7,8,19–21].

**Sample size calculation**

Adequacy of our sample size for the evaluation of PMI as a prognostic factor was determined following the methodology of Schmoor et al. [22]. We assumed an odds ratio of 1.5 of PMI for MACE and all-cause mortality according to previous studies [6,23]. The prevalence of PMI was 0.192 in the study population. The two-sided alpha was 0.05, power was 90%, and a variance inflation factor (VIF) was calculated using McFadden’s R^2^ derived from multivariable logistic regression including all prespecified baseline covariates and PMI as dependent variable. This resulted in a minimum of 330 events for MACE and all-cause mortality. The cohort was therefore deemed sufficient for evaluation of PMI as a prognostic factor.

The maximum number of degrees of freedom relative to the sample size was determined using the *pmsampsize* package. Based on a shrinkage factor of 0.90, and a desired model complexity of approximately 40 degrees of freedom (reflecting the inclusion of spline-transformed continuous predictors and categorical variables), the minimum sample size required for new model development was 3069 with 481 events for all-cause death and 3069 with 482 events for MACE (assuming an outcome prevalence = 0.1567 for death and 0.1569 for MACE) and an events per predictor ratio of 12.02 and 12.04 respectively [24].
