# Supplementary material for: Prevalence and prognostic relevance of perioperative myocardial injury/infarction after major noncardiac surgery in older patients
Source: Age Ageing. 2026 Apr 20;55(4):afag103. doi: 10.1093/ageing/afag103 (PMC13092811; doi:10.1093/ageing/afag103)
Supplement: Appendix_3_afag103 [file appendix_3_afag103.docx]

**Appendix 3: Exploratory Survey of board-certified geriatricians**

**Brief introduction to the study**

Older patients with a high burden of comorbidities are increasingly undergoing major non-cardiac surgery. While in younger patients perioperative myocardial infarction/injury (PMI) is considered prognostically relevant, it remains unclear in older patients with numerous competing comorbidities whether PMI truly has a significant impact on long-term prognosis (1-year mortality and acute cardiac events).

The aim of this survey is to gather the expectations of geriatricians on this issue. Therefore, we rely on your expert opinion as specialists.

**Important definitions**

• Older patients: Age ≥ 70 years with at least 3 comorbidities, or age ≥ 80 years.

• PMI (perioperative myocardial infarction/injury): An increase in the cardiac biomarker troponin above the upper reference limit (hs-cTnT ≥ 14 ng/L), occurring within 3 days after surgery and indicating myocardial injury. This definition applies regardless of whether patients experience symptoms or show ECG changes.

**Questions**

1) Do you think that PMI has a relevant independent impact on 1-year all-cause mortality in older patients?

☐ Yes

☐ No

2) Do you think that PMI has a relevant independent impact on acute cardiac events (acute heart failure, life-threatening arrhythmia, myocardial infarction, cardiovascular death) in older patients?

☐ Yes

☐ No

Questions in original survey were posed in German. Results show answers of geriatricians’ expert opinion on questions, whether or not PMI had prognostic relevance on 1-year all-cause mortality and 1-year MACE, respectively (n = 14)
