# Supplementary material for: Prevalence and prognostic relevance of perioperative myocardial injury/infarction after major noncardiac surgery in older patients
Source: Age Ageing. 2026 Apr 20;55(4):afag103. doi: 10.1093/ageing/afag103 (PMC13092811; doi:10.1093/ageing/afag103)
Supplement: Appendix_4_afag103 [file appendix_4_afag103.docx]

**Appendix 4: Directed acyclic graph (DAG)**
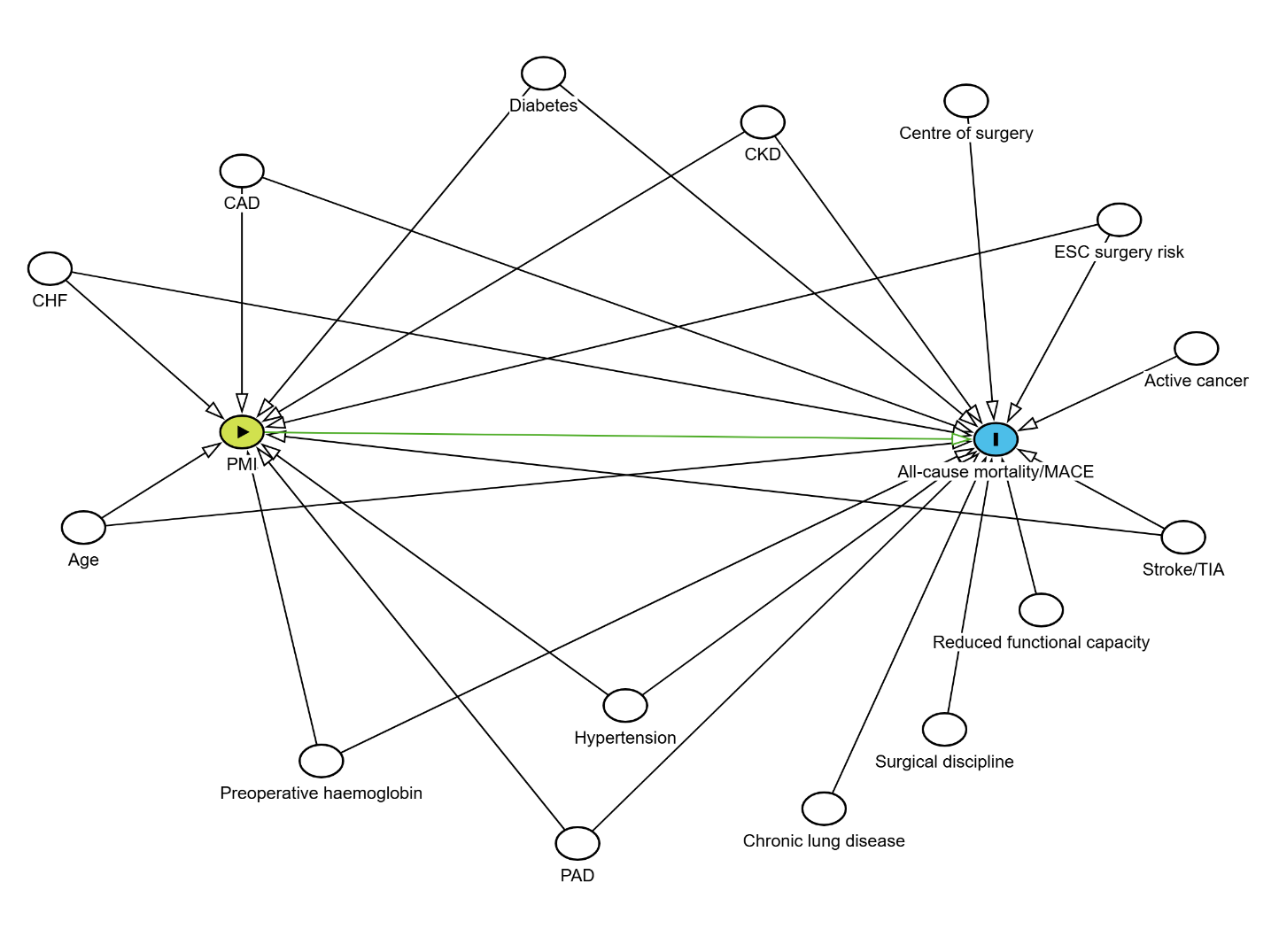


Exposure is PMI, outcome death/MACE. Abbreviations: PAD – peripheral artery disease, CHF – chronic heart failure, CKD – chronic kidney disease, CAD – coronary artery disease, TIA – transient ischaemic attack
