# Supplementary material for: Prevalence and prognostic relevance of perioperative myocardial injury/infarction after major noncardiac surgery in older patients
Source: Age Ageing. 2026 Apr 20;55(4):afag103. doi: 10.1093/ageing/afag103 (PMC13092811; doi:10.1093/ageing/afag103)
Supplement: Appendix_5_afag103 [file appendix_5_afag103.docx]

**Appendix 5: R packages and versions**

| R Package | Version |
| --- | --- |
| adjustedCurves | 0.11.3 |
| broom | 1.0.10 |
| cmprsk | 2.2-12 |
| compareGroups | 4.10.0 |
| consort | 1.2.2 |
| dagitty | 0.3-4 |
| dbplyr | 2.5.1 |
| dplyr | 1.1.4 |
| flexsurv | 2.3.2 |
| flextable | 0.9.10 |
| ggdag | 0.2.13 |
| ggplot2 | 4.0.0 |
| ggraph | 2.2.2 |
| ggsurvfit | 1.2.0 |
| ggtext | 0.1.2 |
| glue | 1.8.0 |
| gridExtra | 2.3 |
| gtsummary | 2.4.0 |
| haven | 2.5.5 |
| Hmisc | 5.2-3 |
| lubridate | 1.9.4 |
| officer | 0.7.0 |
| patchwork | 1.3.2 |
| pmsampsize | 1.1.3 |
| png | 0.1-8 |
| readr | 2.1.5 |
| renv | 1.1.5 |
| rms | 8.0-0 |
| rstpm2 | 1.7.0 |
| scales | 1.4.0 |
| stdReg | 3.4.2 |
| summarytools | 1.1.4 |
| survminer | 0.5.1 |
| survRM3 | 1.0-4 |
| tableone | 0.13.2 |
| tibble | 3.3.0 |
| tidycmprsk | 1.1.0 |
| tidygraph | 1.3.1 |
| tidyr | 1.3.1 |
| tidyverse | 2.0.0 |
| timereg | 2.0.7 |
