# Supplementary material for: Prevalence and prognostic relevance of perioperative myocardial injury/infarction after major noncardiac surgery in older patients
Source: Age Ageing. 2026 Apr 20;55(4):afag103. doi: 10.1093/ageing/afag103 (PMC13092811; doi:10.1093/ageing/afag103)
Supplement: Appendix_6_afag103 [file appendix_6_afag103.docx]

**Appendix 6: Patient flowchart**

**
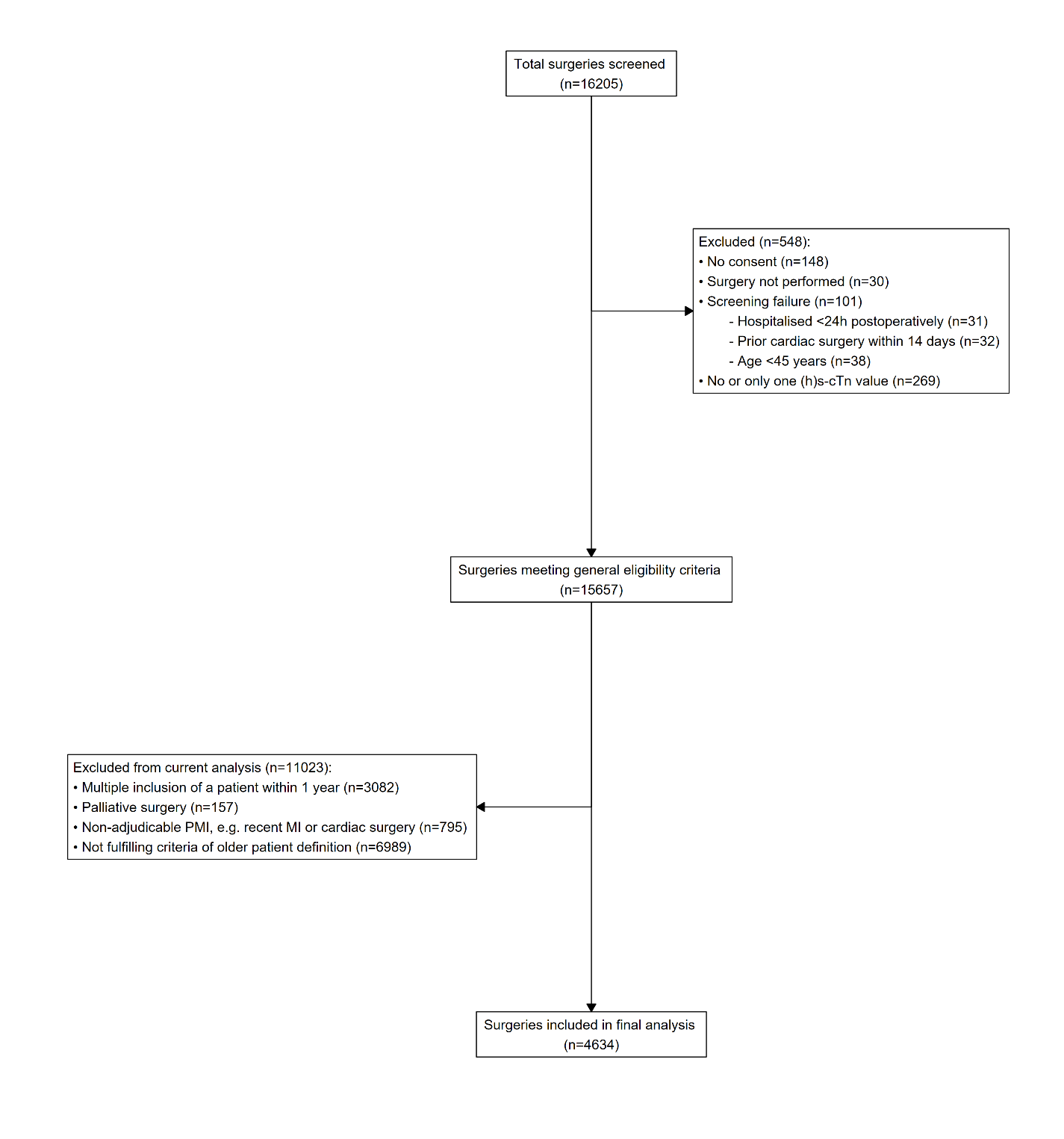
**

Abbreviations: (h)s-cTn – high-sensitivity/sensitive cardiac troponin. Older patients were defined as either ≥70 years of age with ≥3 comorbidities, or ≥80 years regardless of multimorbidity.
