# Supplementary material for: Prevalence and prognostic relevance of perioperative myocardial injury/infarction after major noncardiac surgery in older patients
Source: Age Ageing. 2026 Apr 20;55(4):afag103. doi: 10.1093/ageing/afag103 (PMC13092811; doi:10.1093/ageing/afag103)
Supplement: Appendix_7_afag103 [file appendix_7_afag103.docx]

**Appendix 7: Sensitivity analysis: Endpoint Table with PMI aetiologies in younger population**

| **Endpoints** | **Overall** | **Extracardiac** | **T1MI** | **Tachy-arrhythmia** | **AHF** | **Likely T2MI** |
| --- | --- | --- | --- | --- | --- | --- |
| n (%) | 751 | 117 (15.6) | 52 (6.9) | 26 (3.5) | 14 (1.9) | 542 (72.2) |
| **All-cause death, n (%)** | **124 (16.8)** | **50 (43.1)** | **5 (10.0)** | **6 (23.1)** | **3 (23.1)** | **60 (11.3)** |
| Cardiovascular death, n (%) | 57 (7.6) | 26 (22.2) | 4 (7.7) | 5 (19.2) | 2 (14.3) | 20 (3.7) |
| Non-cardiovascular death, n (%) | 67 (8.9) | 24 (20.5) | 1 (1.9) | 1 (3.8) | 1 (7.1) | 40 (7.4) |
| **MACE without CV-death, n (%)** | **93 (12.4)** | **23 (19.8)** | **13 (25.0)** | **9 (34.6)** | **4 (28.6)** | **44 (8.1)** |
| Acute myocardial infarction, n (%) | 39 (5.2) | 3 (2.6) | 14 (26.9) | 1 (3.8) | 0 (0.0) | 21 (3.9) |
| Acute heart failure, n (%) | 53 (7.1) | 14 (12.0) | 3 (5.8) | 5 (19.2) | 4 (28.6) | 27 (5.0) |
| Life-threatening arrhythmia, n (%) | 28 (3.7) | 10 (8.5) | 4 (7.7) | 3 (11.5) | 1 (7.1) | 10 (1.8) |

Number and percentage of all-cause death and MACE at 1 year in younger patients with PMI according to different PMI aetiologies. MACE defined as composite endpoint; therefore, total number of individual events exceeds total number of composite endpoints. Abbreviations: MACE – major adverse cardiac events, CV-death – cardiovascular death, T1MI – Type 1 myocardial infarction, AHF – acute heart failure, likely T2MI – likely type 2 myocardial infarction.
