# Supplementary material for: Prevalence and prognostic relevance of perioperative myocardial injury/infarction after major noncardiac surgery in older patients
Source: Age Ageing. 2026 Apr 20;55(4):afag103. doi: 10.1093/ageing/afag103 (PMC13092811; doi:10.1093/ageing/afag103)
Supplement: Appendix_9_afag103 [file appendix_9_afag103.docx]

**Appendix 9: Adjusted hazard ratio for 1-year all-cause mortality**

| **Variable** | **Adjusted HR (aHR)** | **P-value** |
| --- | --- | --- |
| **Continuous variables** |  |  |
| Age, per year (2 splines) | 1.03 (0.99–1.06)(1.02 (0.99–1.05)) | 0.174(0.207) |
| Preoperative Hb (2 splines) | 0.98 (0.97–0.98)(1.01 (1–1.02)) | <0.001(0.118) |
| **Surgical discipline** |  |  |
| Ortho/Trauma | Reference |  |
| Neurosurgery | 3.24 (1.19–8.77) | 0.021 |
| Other | 1.92 (1.09–3.37) | 0.024 |
| Spinal | 0.8 (0.58–1.11) | 0.177 |
| Thoracic | 1.18 (0.84–1.66) | 0.33 |
| Urology | 0.81 (0.57–1.14) | 0.221 |
| Vascular | 0.91 (0.68–1.23) | 0.552 |
| Visceral | 0.84 (0.63–1.13) | 0.253 |
| **Centre of surgery** |  |  |
| University hospital Switzerland | Reference |  |
| Cantonal hospital Switzerland | 1.22 (0.95–1.56) | 0.116 |
| University hospital Brazil | 3.07 (2.18–4.33) | <0.001 |
| **ESC Surgery Risk** |  |  |
| ESC Surgery Risk <1% | Reference |  |
| ESC Surgery Risk 1–5% | 0.98 (0.78–1.22) | 0.848 |
| ESC Surgery Risk >5% | 1.63 (1.24–2.15) | <0.001 |
| **Comorbidities** |  |  |
| Active cancer | 2.45 (1.91–3.14) | <0.001 |
| Chronic kidney disease | 1.27 (1.09–1.49) | 0.003 |
| Chronic lung disease | 1.26 (1.05–1.51) | 0.013 |
| Coronary artery disease | 0.97 (0.83–1.15) | 0.753 |
| Diabetes mellitus | 1.04 (0.88–1.22) | 0.63 |
| Chronic heart failure | 1.48 (1.25–1.75) | <0.001 |
| History of Stroke/TIA | 1.02 (0.83–1.25) | 0.862 |
| Hypertension | 0.79 (0.66–0.95) | 0.014 |
| Peripheral artery disease | 1.07 (0.86–1.32) | 0.568 |
| Reduced functional capacity | 1.89 (1.56–2.3) | <0.001 |

Calculation with timevarying cox-regression model. Abbreviations: Hb – haemoglobin, TIA – transient ischaemic attack.
