# Supplementary material for: Prevalence and prognostic relevance of perioperative myocardial injury/infarction after major noncardiac surgery in older patients
Source: Age Ageing. 2026 Apr 20;55(4):afag103. doi: 10.1093/ageing/afag103 (PMC13092811; doi:10.1093/ageing/afag103)
Supplement: Appendix_10_afag103 [file appendix_10_afag103.docx]

**Appendix 10: Subdistribution hazard ratio timepoints MACE**

**
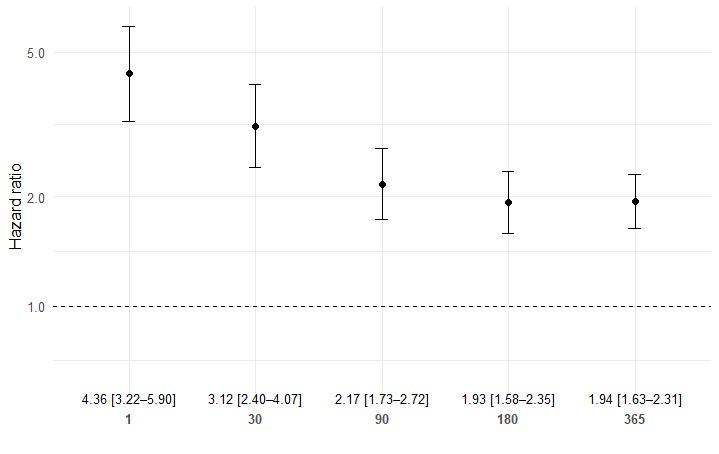
**

X-axis shows days after surgery. Maximum follow-up days 365 days. Hazard ratios and confidence intervals shown above x-axis in graph.
