# Supplementary material for: Prevalence and prognostic relevance of perioperative myocardial injury/infarction after major noncardiac surgery in older patients
Source: Age Ageing. 2026 Apr 20;55(4):afag103. doi: 10.1093/ageing/afag103 (PMC13092811; doi:10.1093/ageing/afag103)
Supplement: Appendix_11_afag103 [file appendix_11_afag103.docx]

**Appendix 11: Adjusted hazard ratio for 1-year MACE**

| **Variable** | **Adjusted HR (aHR)** | **P-value** |
| --- | --- | --- |
| **Continuous variables** |  |  |
| Age, per year (2 splines) | 1.44 (0.97–2.13) (2.12 (0.98–4.61); 2.25 (1.06–4.76)) | 0.067 (0.057; 0.034) |
| Preoperative Hb (2 splines) | 0.43 (0.29–0.63) (0.31 (0.09–1.12); 0.49 (0.15–1.61)) | <0.001 (0.074; 0.238) |
| **Surgical discipline** |  |  |
| Ortho/Trauma | Reference |  |
| Spinal | 0.9 (0.65–1.25) | 0.534 |
| Thoracic | 0.89 (0.57–1.4) | 0.618 |
| Visceral | 0.84 (0.59–1.19) | 0.328 |
| Urology | 0.94 (0.64–1.39) | 0.774 |
| Neurosurgery | 0.7 (0.14–3.54) | 0.667 |
| Vascular | 0.65 (0.46–0.92) | 0.015 |
| Other | 1.74 (0.88–3.44) | 0.111 |
| **Centre of surgery** |  |  |
| University hospital Switzerland | Reference |  |
| Cantonal hospital Switzerland | 1.4 (1.06–1.86) | 0.019 |
| University hospital Brazil | 2.52 (1.61–3.94) | <0.001 |
| **ESC Surgery Risk** |  |  |
| ESC Surgery Risk <1% | Reference |  |
| ESC Surgery Risk 1–5% | 0.95 (0.74–1.23) | 0.714 |
| ESC Surgery Risk >5% | 1.39 (0.98–1.97) | 0.062 |
| **Comorbidities** |  |  |
| Chronic heart failure | 1.98 (1.65–2.39) | <0.001 |
| Hypertension | 0.92 (0.73–1.15) | 0.458 |
| Peripheral artery disease | 1.2 (0.95–1.53) | 0.132 |
| History of Stroke/TIA | 1.04 (0.82–1.32) | 0.734 |
| Chronic kidney disease | 1.07 (0.88–1.28) | 0.503 |
| Active cancer | 0.92 (0.66–1.27) | 0.611 |
| Coronary artery disease | 1.28 (1.05–1.55) | 0.012 |
| Diabetes mellitus | 1.18 (0.98–1.42) | 0.082 |
| Chronic lung disease | 1.28 (1.03–1.59) | 0.025 |
| Reduced functional capacity | 1.66 (1.31–2.12) | <0.001 |

Calculation with subdistributional hazard model. Abbreviations: Hb – haemoglobin, TIA – transient ischaemic attack.
