# Supplementary material for: Prevalence and prognostic relevance of perioperative myocardial injury/infarction after major noncardiac surgery in older patients
Source: Age Ageing. 2026 Apr 20;55(4):afag103. doi: 10.1093/ageing/afag103 (PMC13092811; doi:10.1093/ageing/afag103)
Supplement: Appendix_12_afag103 [file appendix_12_afag103.docx]

**Appendix 12: Sensitivity analysis for A Cumulative incidence of all-cause mortality and B Cumulative incidence of MACE**

**
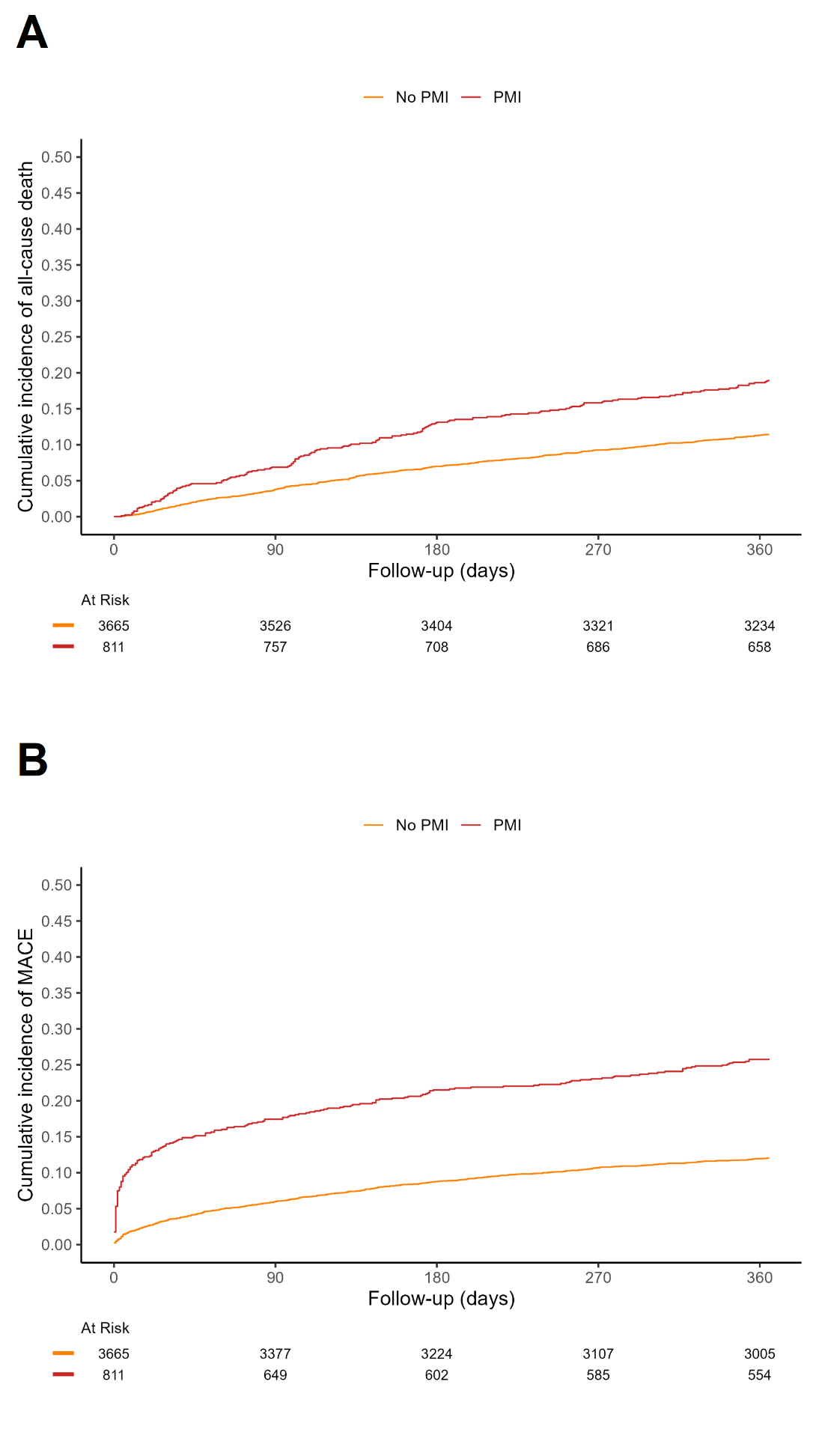
**

For sensitivity analysis, the cohort was filtered for patients alive after hospital discharge. A total of 101 patients were excluded from analysis due to missing follow-up data.
