# Supplementary material for: Prevalence and prognostic relevance of perioperative myocardial injury/infarction after major noncardiac surgery in older patients
Source: Age Ageing. 2026 Apr 20;55(4):afag103. doi: 10.1093/ageing/afag103 (PMC13092811; doi:10.1093/ageing/afag103)
Supplement: Appendix_13_afag103 [file appendix_13_afag103.docx]

**Appendix 13: Sensitivity analysis for A Cause-specific hazard ratio of all-cause mortality and B Subdistribution hazard ratio of MACE in geriatric profile patients independent of age**

**
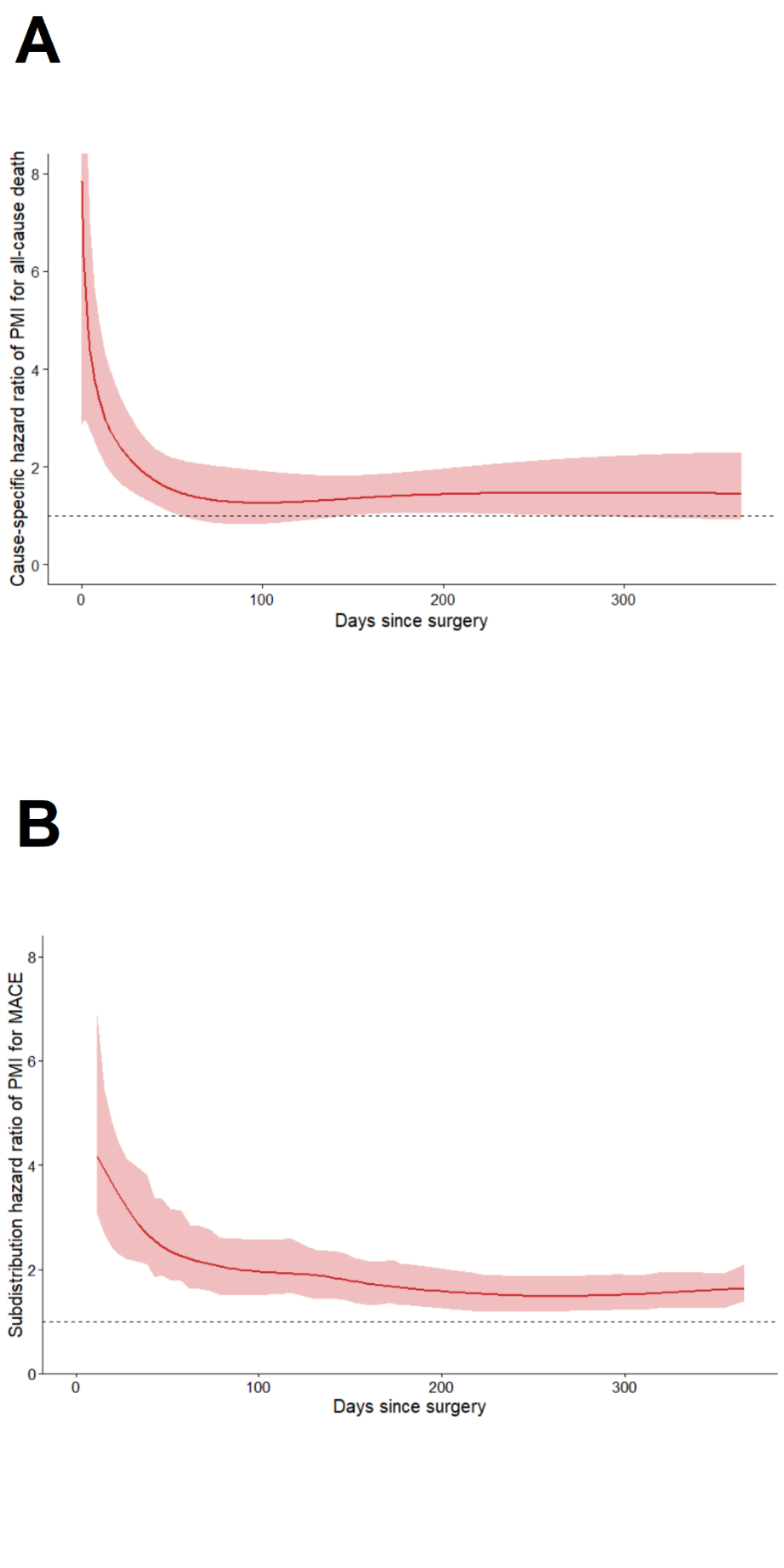
**

For sensitivity analysis, the geriatric status patients were defined independent of age. Geriatric status was defined as having ≥3 comorbidities and reduced functional capacity (<4 metabolic equivalent tasks). N = 2080.
