# Supplementary material for: Prevalence and prognostic relevance of perioperative myocardial injury/infarction after major noncardiac surgery in older patients
Source: Age Ageing. 2026 Apr 20;55(4):afag103. doi: 10.1093/ageing/afag103 (PMC13092811; doi:10.1093/ageing/afag103)
Supplement: Appendix_14_afag103 [file appendix_14_afag103.docx]

**Appendix 14: Sensitivity analyses for A Cause-specific hazard ratio of all-cause mortality and B Subdistribution hazard ratio of MACE in age cut-offs regardless of comorbidities at I) Cut-off at 60 years, II) Cut-off at 70 years, III) Cut-off at 80 years**

1. **
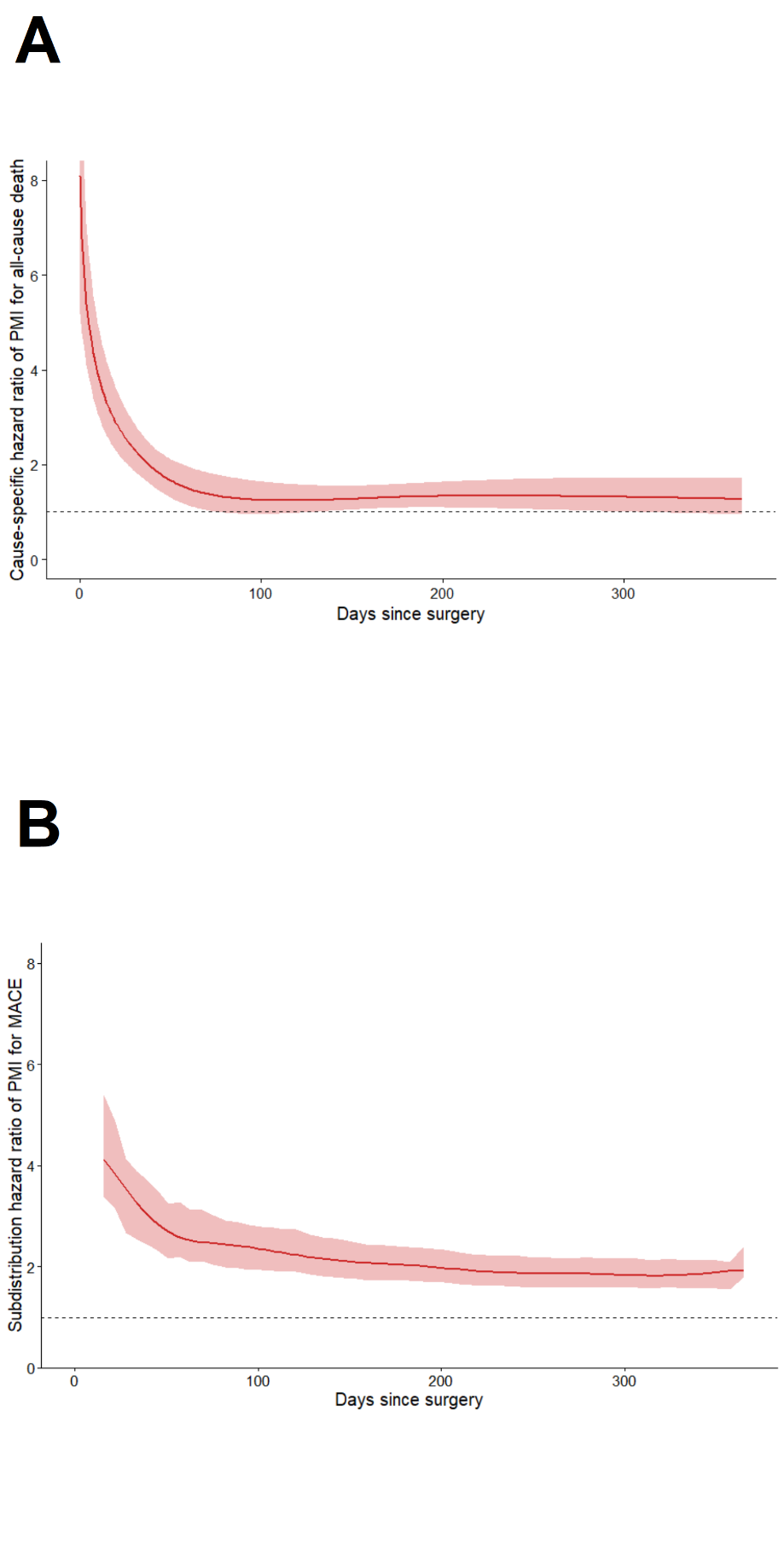
**Cut-off at 60 years (n = 10924)
2. Cut-off at 70 years (n = 7814)

**
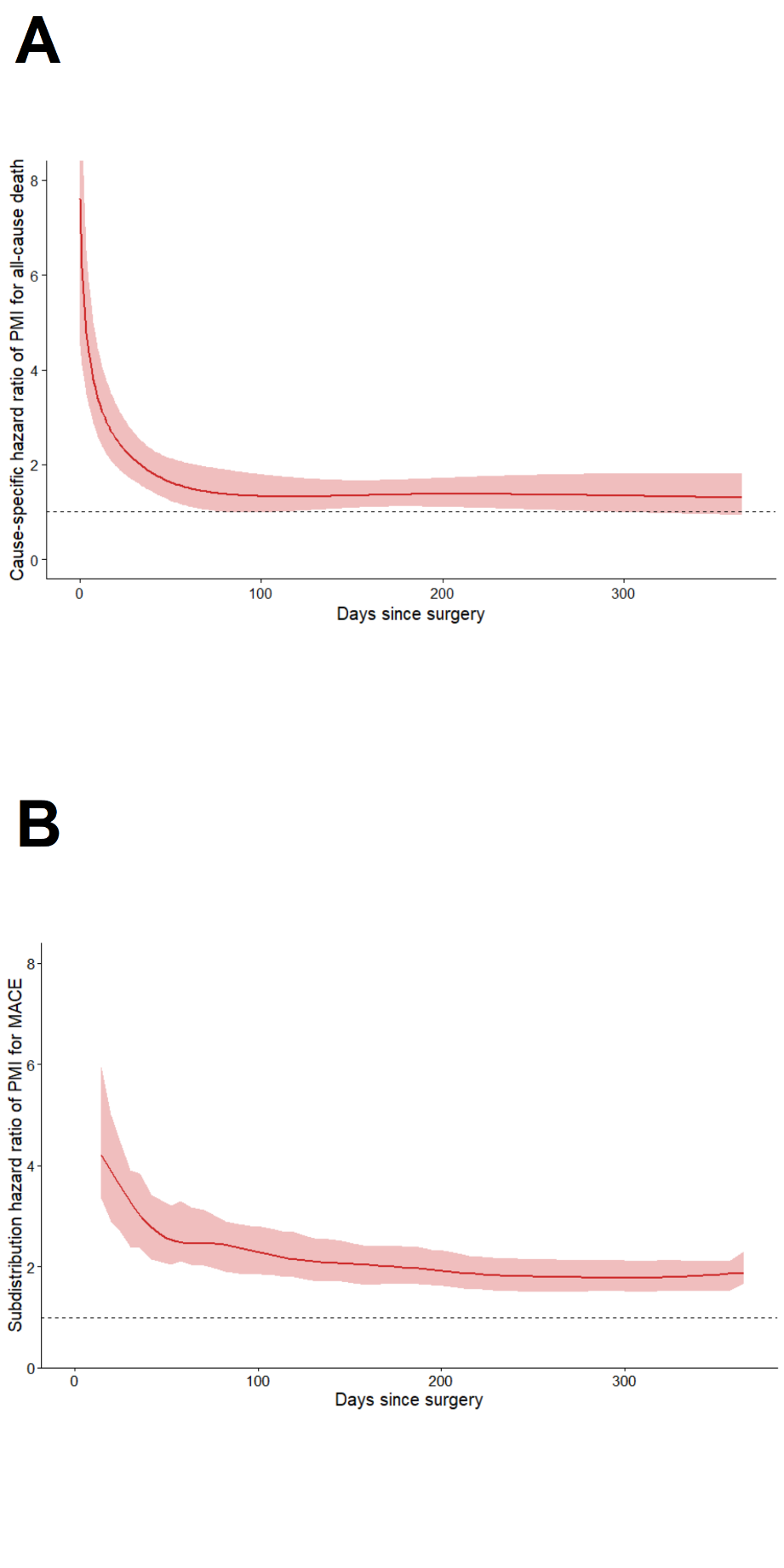
**

1.
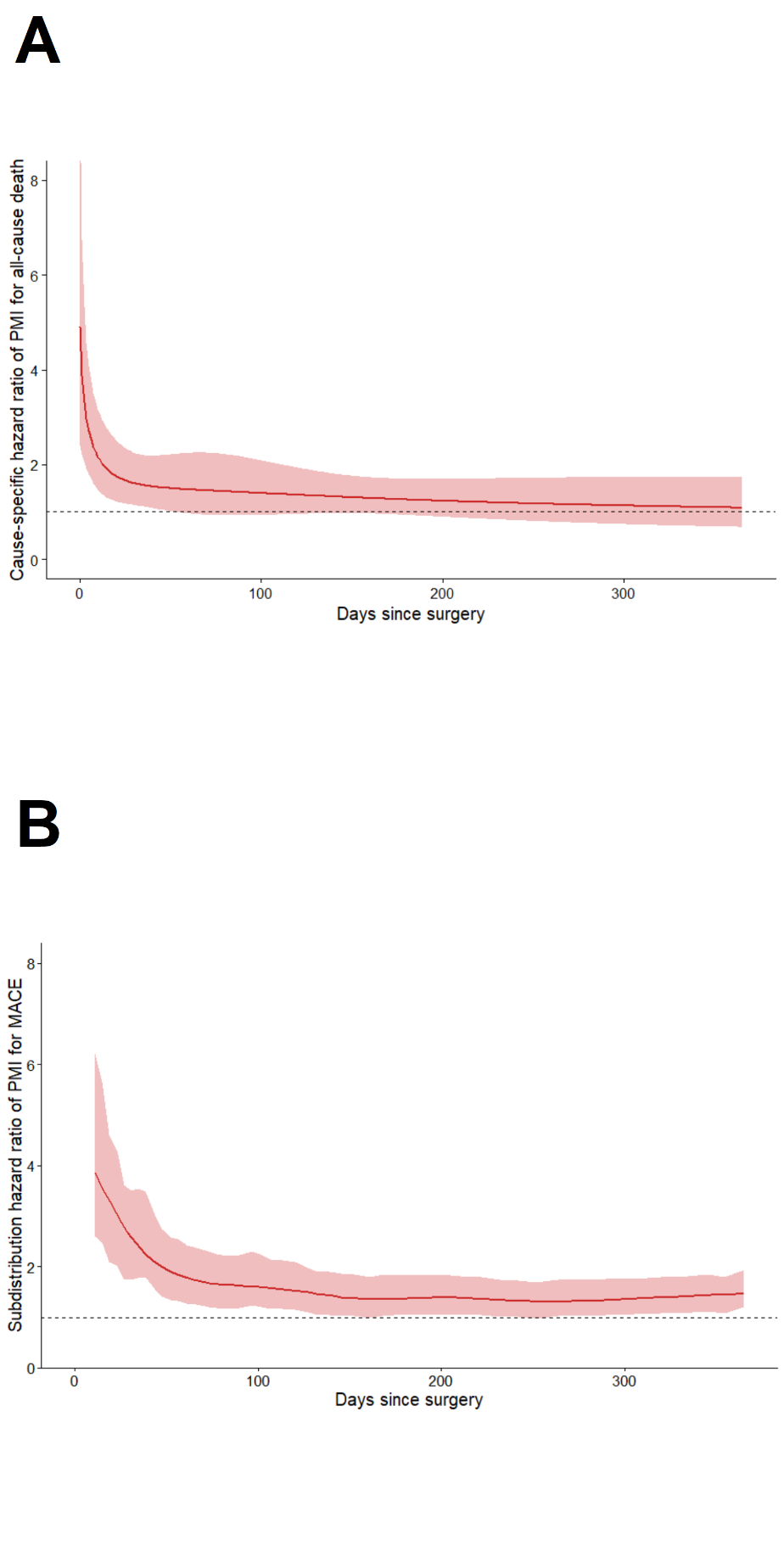
Cut-off at 80 years (n = 2641)
