# Supplementary material for: Prevalence and prognostic relevance of perioperative myocardial injury/infarction after major noncardiac surgery in older patients
Source: Age Ageing. 2026 Apr 20;55(4):afag103. doi: 10.1093/ageing/afag103 (PMC13092811; doi:10.1093/ageing/afag103)
Supplement: Appendix_15_afag103 [file appendix_15_afag103.docx]

**Appendix 15: Results of exploratory survey of board-certified geriatricians**

**
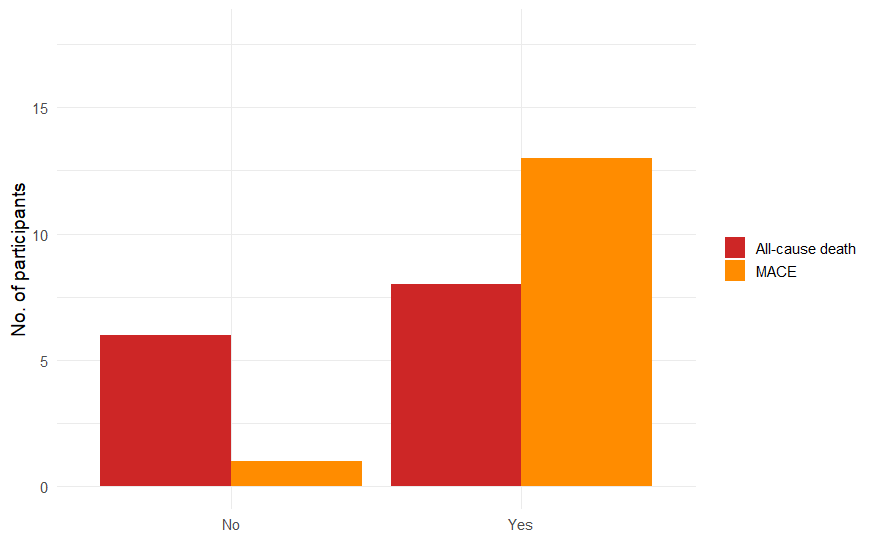
**

Results of the exploratory survey: Among the 14 board-certified geriatricians completing the survey, 6/14 (43%) did not consider PMI to be an independent predictor of all-cause mortality at 1 year. In contrast, nearly all 13/14 (93%) assumed that PMI was an independent predictor of MACE at 1 year.
