# Supplementary material for: Prevalence and prognostic relevance of perioperative myocardial injury/infarction after major noncardiac surgery in older patients
Source: Age Ageing. 2026 Apr 20;55(4):afag103. doi: 10.1093/ageing/afag103 (PMC13092811; doi:10.1093/ageing/afag103)
Supplement: aa-25-3712-File002_afag103 [file aa-25-3712-file002_afag103.docx]

**Prevalence and prognostic relevance of perioperative myocardial injury/infarction after major noncardiac surgery in older patients**

**Appendices: Contents**

**Appendix 1:** STROBE checklist

**Appendix 2:** Supplementary Methods

**Appendix 3:** Exploratory survey of board-certified geriatricians

**Appendix 4:** Directed acyclic graph (DAG)

**Appendix 5:** R packages and versions

**Appendix 6:** Patient flowchart

**Appendix 7:** Sensitivity analysis: Endpoint Table with PMI aetiologies in younger population

**Appendix 8:** Cause-specific hazard ratio timepoints all-cause mortality

**Appendix 9:** Adjusted hazard ratio for 1-year all-cause mortality

**Appendix 10:** Subdistribution hazard ratio timepoints MACE

**Appendix 11:** Adjusted hazard ratio for 1-year MACE

**Appendix 12:** Sensitivity analysis for A Cumulative incidence of all-cause death and B Cumulative incidence of MACE

**Appendix 13:** Sensitivity analysis for A Cause-specific hazard ratio of all-cause mortality and B Subdistribution hazard ratio of MACE in geriatric profile patients independent of age

**Appendix 14:** Sensitivity analyses for A Cause-specific hazard ratio of all-cause mortality and B Subdistribution hazard ratio of MACE in age cut-offs regardless of comorbidities at I) Cut-off at 60 years, II) Cut-off at 70 years, III) Cut-off at 80 years

**Appendix 15:** Results of exploratory survey of board-certified geriatricians

**Appendix 1: STROBE checklist**

STROBE Statement—checklist of items that should be included in reports of observational studies [1]

|  | Item No | Recommendation |
| --- | --- | --- |
| **Title and abstract** | 1 | (*a*) Indicate the study’s design with a commonly used term in the title or the abstract |
|  |  | (*b*) Provide in the abstract an informative and balanced summary of what was done and what was found |
| Introduction | | |
| Background/rationale | 2 | Explain the scientific background and rationale for the investigation being reported |
| Objectives | 3 | State specific objectives, including any prespecified hypotheses |
| Methods | | |
| Study design | 4 | Present key elements of study design early in the paper |
| Setting | 5 | Describe the setting, locations, and relevant dates, including periods of recruitment, exposure, follow-up, and data collection |
| Participants | 6 | (*a*) *Cohort study*—Give the eligibility criteria, and the sources and methods of selection of participants. Describe methods of follow-up  *Case-control study*—Give the eligibility criteria, and the sources and methods of case ascertainment and control selection. Give the rationale for the choice of cases and controls  *Cross-sectional study*—Give the eligibility criteria, and the sources and methods of selection of participants |
|  |  | (*b*) *Cohort study*—For matched studies, give matching criteria and number of exposed and unexposed  *Case-control study*—For matched studies, give matching criteria and the number of controls per case |
| Variables | 7 | Clearly define all outcomes, exposures, predictors, potential confounders, and effect modifiers. Give diagnostic criteria, if applicable |
| Data sources/ measurement | 8* | For each variable of interest, give sources of data and details of methods of assessment (measurement). Describe comparability of assessment methods if there is more than one group |
| Bias | 9 | Describe any efforts to address potential sources of bias |
| Study size | 10 | Explain how the study size was arrived at |
| Quantitative variables | 11 | Explain how quantitative variables were handled in the analyses. If applicable, describe which groupings were chosen and why |
| Statistical methods | 12 | (*a*) Describe all statistical methods, including those used to control for confounding |
|  |  | (*b*) Describe any methods used to examine subgroups and interactions |
|  |  | (*c*) Explain how missing data were addressed |
|  |  | (*d*) *Cohort study*—If applicable, explain how loss to follow-up was addressed  *Case-control study*—If applicable, explain how matching of cases and controls was addressed  *Cross-sectional study*—If applicable, describe analytical methods taking account of sampling strategy |
|  |  | (*e*) Describe any sensitivity analyses |

| Results | | |
| --- | --- | --- |
| Participants | 13* | (a) Report numbers of individuals at each stage of study—eg numbers potentially eligible, examined for eligibility, confirmed eligible, included in the study, completing follow-up, and analysed |
|  |  | (b) Give reasons for non-participation at each stage |
|  |  | (c) Consider use of a flow diagram |
| Descriptive data | 14* | (a) Give characteristics of study participants (eg demographic, clinical, social) and information on exposures and potential confounders |
|  |  | (b) Indicate number of participants with missing data for each variable of interest |
|  |  | (c) *Cohort study*—Summarise follow-up time (eg, average and total amount) |
| Outcome data | 15* | *Cohort study*—Report numbers of outcome events or summary measures over time |
|  |  | *Case-control study—*Report numbers in each exposure category, or summary measures of exposure |
|  |  | *Cross-sectional study—*Report numbers of outcome events or summary measures |
| Main results | 16 | (*a*) Give unadjusted estimates and, if applicable, confounder-adjusted estimates and their precision (eg, 95% confidence interval). Make clear which confounders were adjusted for and why they were included |
|  |  | (*b*) Report category boundaries when continuous variables were categorized |
|  |  | (*c*) If relevant, consider translating estimates of relative risk into absolute risk for a meaningful time period |
| Other analyses | 17 | Report other analyses done—eg analyses of subgroups and interactions, and sensitivity analyses |
| Discussion | | |
| Key results | 18 | Summarise key results with reference to study objectives |
| Limitations | 19 | Discuss limitations of the study, taking into account sources of potential bias or imprecision. Discuss both direction and magnitude of any potential bias |
| Interpretation | 20 | Give a cautious overall interpretation of results considering objectives, limitations, multiplicity of analyses, results from similar studies, and other relevant evidence |
| Generalisability | 21 | Discuss the generalisability (external validity) of the study results |
| Other information | | |
| Funding | 22 | Give the source of funding and the role of the funders for the present study and, if applicable, for the original study on which the present article is based |

*Give information separately for cases and controls in case-control studies and, if applicable, for exposed and unexposed groups in cohort and cross-sectional studies.

**Note:** An Explanation and Elaboration article discusses each checklist item and gives methodological background and published examples of transparent reporting. The STROBE checklist is best used in conjunction with this article (freely available on the Web sites of PLoS Medicine at http://www.plosmedicine.org/, Annals of Internal Medicine at http://www.annals.org/, and Epidemiology at http://www.epidem.com/). Information on the STROBE Initiative is available at www.strobe-statement.org.

**Appendix 2: Supplementary Methods**

**Population**

Patients were eligible for the institutional active PMI surveillance and included if they were considered at increased cardiovascular risk, defined as ≥65 years of age or ≥45 years with a history of coronary artery disease (CAD), peripheral artery disease (PAD), or stroke/transient ischaemic attack (TIA), undergoing major inpatient noncardiac surgery with a planned postoperative stay of ≥24 h [2–8].

The definition of older patients was established according to 1) the Swiss Frailty Network and Repository, other European societies and local standards [9–14], and 2) based on meta-analyses showing increased prevalence of frailty and research showing an increase in morbidity and mortality burden at ≥70 years of age [15–18].

**PMI definition**

PMI aetiology was centrally adjudicated by two independent experts based on all available clinical data, including ECG, serial laboratory measurements including cTn and haemoglobin, vital sign monitoring in the peri- and intraoperative period, and echocardiography, cardiac stress testing, and coronary angiography if performed. In cases of disagreement between the two adjudicating reviewers, consensus was sought and found by discussion with a third reviewer. PMI was hierarchically classified based on the likely trigger for myocardial injury or infarction, aiming to reflect different clinical management pathways [7,8,19,20]. Following classification was applied: (1) extracardiac if caused by a primarily extracardiac disease such as severe sepsis, stroke, pulmonary embolism, or blunt or surgical cardiac trauma; (2) cardiac, further subtyped into type 1 myocardial infarction (T1MI), tachyarrhythmia, or AHF; (3) cardiac, likely type 2 myocardial infarction (LT2MI) if the causes mentioned above (1, 2) could be ruled out and additionally a documented or suspected type 2 trigger (e.g. severe hypotension, anaemia, hypoxia, sinus tachycardia) was present [7,8,19–21].

**Sample size calculation**

Adequacy of our sample size for the evaluation of PMI as a prognostic factor was determined following the methodology of Schmoor et al. [22]. We assumed an odds ratio of 1.5 of PMI for MACE and all-cause mortality according to previous studies [6,23]. The prevalence of PMI was 0.192 in the study population. The two-sided alpha was 0.05, power was 90%, and a variance inflation factor (VIF) was calculated using McFadden’s R^2^ derived from multivariable logistic regression including all prespecified baseline covariates and PMI as dependent variable. This resulted in a minimum of 330 events for MACE and all-cause mortality. The cohort was therefore deemed sufficient for evaluation of PMI as a prognostic factor.

The maximum number of degrees of freedom relative to the sample size was determined using the *pmsampsize* package. Based on a shrinkage factor of 0.90, and a desired model complexity of approximately 40 degrees of freedom (reflecting the inclusion of spline-transformed continuous predictors and categorical variables), the minimum sample size required for new model development was 3069 with 481 events for all-cause death and 3069 with 482 events for MACE (assuming an outcome prevalence = 0.1567 for death and 0.1569 for MACE) and an events per predictor ratio of 12.02 and 12.04 respectively [24].

**Appendix 3: Exploratory Survey of board-certified geriatricians**

**Brief introduction to the study**

Older patients with a high burden of comorbidities are increasingly undergoing major non-cardiac surgery. While in younger patients perioperative myocardial infarction/injury (PMI) is considered prognostically relevant, it remains unclear in older patients with numerous competing comorbidities whether PMI truly has a significant impact on long-term prognosis (1-year mortality and acute cardiac events).

The aim of this survey is to gather the expectations of geriatricians on this issue. Therefore, we rely on your expert opinion as specialists.

**Important definitions**

• Older patients: Age ≥ 70 years with at least 3 comorbidities, or age ≥ 80 years.

• PMI (perioperative myocardial infarction/injury): An increase in the cardiac biomarker troponin above the upper reference limit (hs-cTnT ≥ 14 ng/L), occurring within 3 days after surgery and indicating myocardial injury. This definition applies regardless of whether patients experience symptoms or show ECG changes.

**Questions**

1) Do you think that PMI has a relevant independent impact on 1-year all-cause mortality in older patients?

☐ Yes

☐ No

2) Do you think that PMI has a relevant independent impact on acute cardiac events (acute heart failure, life-threatening arrhythmia, myocardial infarction, cardiovascular death) in older patients?

☐ Yes

☐ No

Questions in original survey were posed in German. Results show answers of geriatricians’ expert opinion on questions, whether or not PMI had prognostic relevance on 1-year all-cause mortality and 1-year MACE, respectively (n = 14)

**Appendix 4: Directed acyclic graph (DAG)**
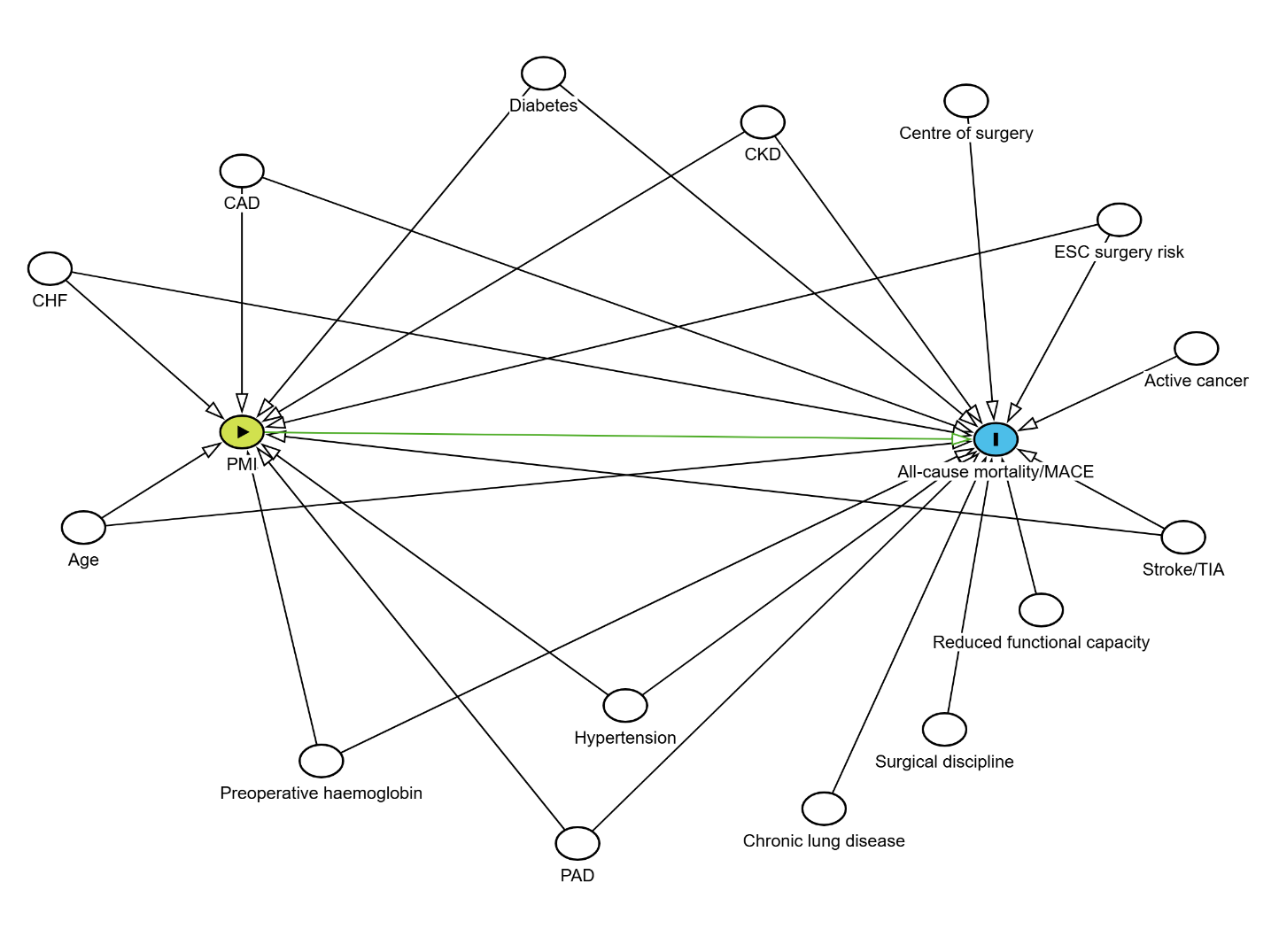


Exposure is PMI, outcome death/MACE. Abbreviations: PAD – peripheral artery disease, CHF – chronic heart failure, CKD – chronic kidney disease, CAD – coronary artery disease, TIA – transient ischaemic attack

**Appendix 5: R packages and versions**

| R Package | Version |
| --- | --- |
| adjustedCurves | 0.11.3 |
| broom | 1.0.10 |
| cmprsk | 2.2-12 |
| compareGroups | 4.10.0 |
| consort | 1.2.2 |
| dagitty | 0.3-4 |
| dbplyr | 2.5.1 |
| dplyr | 1.1.4 |
| flexsurv | 2.3.2 |
| flextable | 0.9.10 |
| ggdag | 0.2.13 |
| ggplot2 | 4.0.0 |
| ggraph | 2.2.2 |
| ggsurvfit | 1.2.0 |
| ggtext | 0.1.2 |
| glue | 1.8.0 |
| gridExtra | 2.3 |
| gtsummary | 2.4.0 |
| haven | 2.5.5 |
| Hmisc | 5.2-3 |
| lubridate | 1.9.4 |
| officer | 0.7.0 |
| patchwork | 1.3.2 |
| pmsampsize | 1.1.3 |
| png | 0.1-8 |
| readr | 2.1.5 |
| renv | 1.1.5 |
| rms | 8.0-0 |
| rstpm2 | 1.7.0 |
| scales | 1.4.0 |
| stdReg | 3.4.2 |
| summarytools | 1.1.4 |
| survminer | 0.5.1 |
| survRM3 | 1.0-4 |
| tableone | 0.13.2 |
| tibble | 3.3.0 |
| tidycmprsk | 1.1.0 |
| tidygraph | 1.3.1 |
| tidyr | 1.3.1 |
| tidyverse | 2.0.0 |
| timereg | 2.0.7 |

**Appendix 6: Patient flowchart**

**
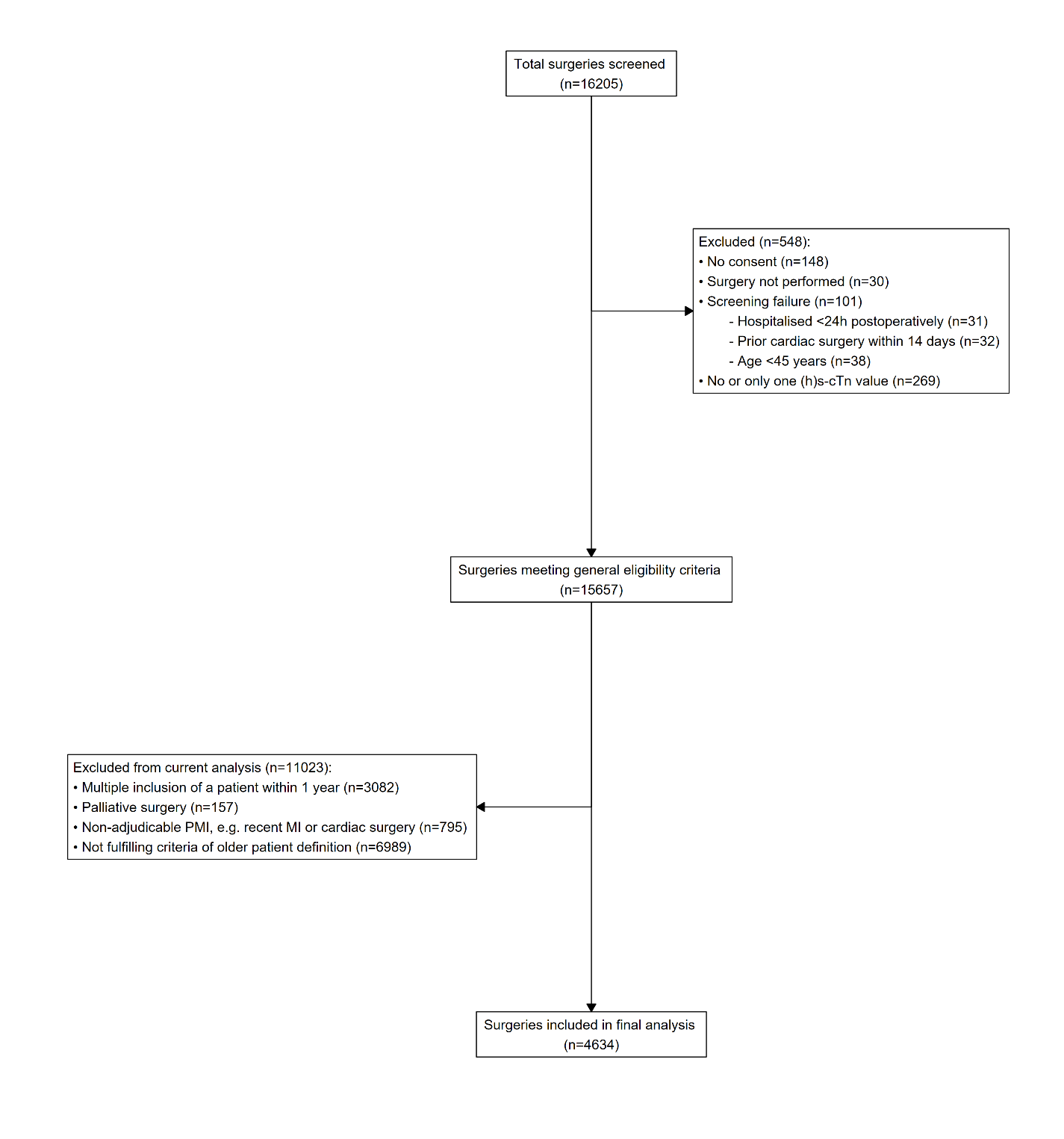
**

Abbreviations: (h)s-cTn – high-sensitivity/sensitive cardiac troponin. Older patients were defined as either ≥70 years of age with ≥3 comorbidities, or ≥80 years regardless of multimorbidity.

**Appendix 7: Sensitivity analysis: Endpoint Table with PMI aetiologies in younger population**

| **Endpoints** | **Overall** | **Extracardiac** | **T1MI** | **Tachy-arrhythmia** | **AHF** | **Likely T2MI** |
| --- | --- | --- | --- | --- | --- | --- |
| n (%) | 751 | 117 (15.6) | 52 (6.9) | 26 (3.5) | 14 (1.9) | 542 (72.2) |
| **All-cause death, n (%)** | **124 (16.8)** | **50 (43.1)** | **5 (10.0)** | **6 (23.1)** | **3 (23.1)** | **60 (11.3)** |
| Cardiovascular death, n (%) | 57 (7.6) | 26 (22.2) | 4 (7.7) | 5 (19.2) | 2 (14.3) | 20 (3.7) |
| Non-cardiovascular death, n (%) | 67 (8.9) | 24 (20.5) | 1 (1.9) | 1 (3.8) | 1 (7.1) | 40 (7.4) |
| **MACE without CV-death, n (%)** | **93 (12.4)** | **23 (19.8)** | **13 (25.0)** | **9 (34.6)** | **4 (28.6)** | **44 (8.1)** |
| Acute myocardial infarction, n (%) | 39 (5.2) | 3 (2.6) | 14 (26.9) | 1 (3.8) | 0 (0.0) | 21 (3.9) |
| Acute heart failure, n (%) | 53 (7.1) | 14 (12.0) | 3 (5.8) | 5 (19.2) | 4 (28.6) | 27 (5.0) |
| Life-threatening arrhythmia, n (%) | 28 (3.7) | 10 (8.5) | 4 (7.7) | 3 (11.5) | 1 (7.1) | 10 (1.8) |

Number and percentage of all-cause death and MACE at 1 year in younger patients with PMI according to different PMI aetiologies. MACE defined as composite endpoint; therefore, total number of individual events exceeds total number of composite endpoints. Abbreviations: MACE – major adverse cardiac events, CV-death – cardiovascular death, T1MI – Type 1 myocardial infarction, AHF – acute heart failure, likely T2MI – likely type 2 myocardial infarction.

**Appendix 8: Cause-specific hazard ratio timepoints all-cause mortality
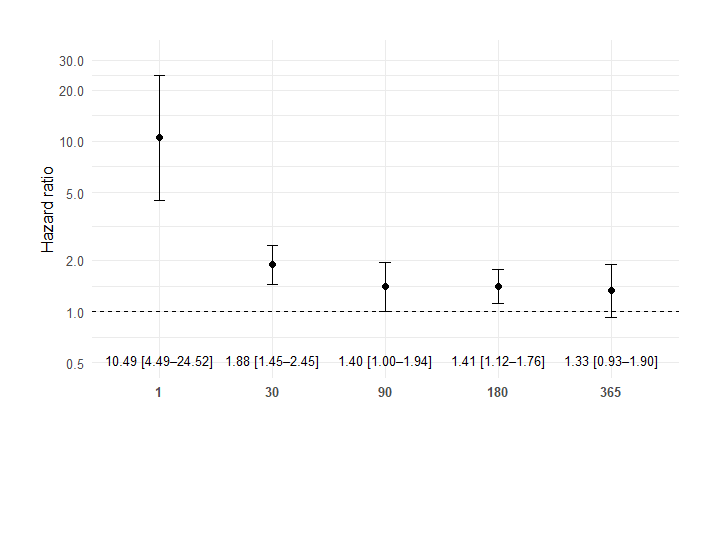
**

X-axis shows days after surgery. Maximum follow-up days 365 days. Hazard ratios and confidence intervals shown above x-axis in graph.

**Appendix 9: Adjusted hazard ratio for 1-year all-cause mortality**

| **Variable** | **Adjusted HR (aHR)** | **P-value** |
| --- | --- | --- |
| **Continuous variables** |  |  |
| Age, per year (2 splines) | 1.03 (0.99–1.06)(1.02 (0.99–1.05)) | 0.174(0.207) |
| Preoperative Hb (2 splines) | 0.98 (0.97–0.98)(1.01 (1–1.02)) | <0.001(0.118) |
| **Surgical discipline** |  |  |
| Ortho/Trauma | Reference |  |
| Neurosurgery | 3.24 (1.19–8.77) | 0.021 |
| Other | 1.92 (1.09–3.37) | 0.024 |
| Spinal | 0.8 (0.58–1.11) | 0.177 |
| Thoracic | 1.18 (0.84–1.66) | 0.33 |
| Urology | 0.81 (0.57–1.14) | 0.221 |
| Vascular | 0.91 (0.68–1.23) | 0.552 |
| Visceral | 0.84 (0.63–1.13) | 0.253 |
| **Centre of surgery** |  |  |
| University hospital Switzerland | Reference |  |
| Cantonal hospital Switzerland | 1.22 (0.95–1.56) | 0.116 |
| University hospital Brazil | 3.07 (2.18–4.33) | <0.001 |
| **ESC Surgery Risk** |  |  |
| ESC Surgery Risk <1% | Reference |  |
| ESC Surgery Risk 1–5% | 0.98 (0.78–1.22) | 0.848 |
| ESC Surgery Risk >5% | 1.63 (1.24–2.15) | <0.001 |
| **Comorbidities** |  |  |
| Active cancer | 2.45 (1.91–3.14) | <0.001 |
| Chronic kidney disease | 1.27 (1.09–1.49) | 0.003 |
| Chronic lung disease | 1.26 (1.05–1.51) | 0.013 |
| Coronary artery disease | 0.97 (0.83–1.15) | 0.753 |
| Diabetes mellitus | 1.04 (0.88–1.22) | 0.63 |
| Chronic heart failure | 1.48 (1.25–1.75) | <0.001 |
| History of Stroke/TIA | 1.02 (0.83–1.25) | 0.862 |
| Hypertension | 0.79 (0.66–0.95) | 0.014 |
| Peripheral artery disease | 1.07 (0.86–1.32) | 0.568 |
| Reduced functional capacity | 1.89 (1.56–2.3) | <0.001 |

Calculation with timevarying cox-regression model. Abbreviations: Hb – haemoglobin, TIA – transient ischaemic attack.

**Appendix 10: Subdistribution hazard ratio timepoints MACE**

**
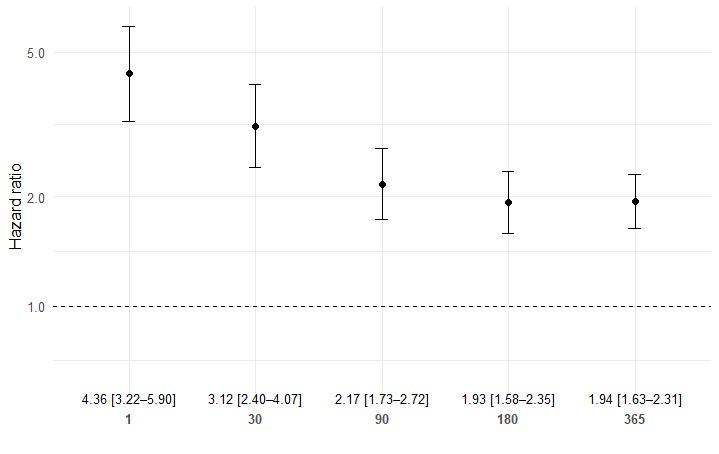
**

X-axis shows days after surgery. Maximum follow-up days 365 days. Hazard ratios and confidence intervals shown above x-axis in graph.

**Appendix 11: Adjusted hazard ratio for 1-year MACE**

| **Variable** | **Adjusted HR (aHR)** | **P-value** |
| --- | --- | --- |
| **Continuous variables** |  |  |
| Age, per year (2 splines) | 1.44 (0.97–2.13) (2.12 (0.98–4.61); 2.25 (1.06–4.76)) | 0.067 (0.057; 0.034) |
| Preoperative Hb (2 splines) | 0.43 (0.29–0.63) (0.31 (0.09–1.12); 0.49 (0.15–1.61)) | <0.001 (0.074; 0.238) |
| **Surgical discipline** |  |  |
| Ortho/Trauma | Reference |  |
| Spinal | 0.9 (0.65–1.25) | 0.534 |
| Thoracic | 0.89 (0.57–1.4) | 0.618 |
| Visceral | 0.84 (0.59–1.19) | 0.328 |
| Urology | 0.94 (0.64–1.39) | 0.774 |
| Neurosurgery | 0.7 (0.14–3.54) | 0.667 |
| Vascular | 0.65 (0.46–0.92) | 0.015 |
| Other | 1.74 (0.88–3.44) | 0.111 |
| **Centre of surgery** |  |  |
| University hospital Switzerland | Reference |  |
| Cantonal hospital Switzerland | 1.4 (1.06–1.86) | 0.019 |
| University hospital Brazil | 2.52 (1.61–3.94) | <0.001 |
| **ESC Surgery Risk** |  |  |
| ESC Surgery Risk <1% | Reference |  |
| ESC Surgery Risk 1–5% | 0.95 (0.74–1.23) | 0.714 |
| ESC Surgery Risk >5% | 1.39 (0.98–1.97) | 0.062 |
| **Comorbidities** |  |  |
| Chronic heart failure | 1.98 (1.65–2.39) | <0.001 |
| Hypertension | 0.92 (0.73–1.15) | 0.458 |
| Peripheral artery disease | 1.2 (0.95–1.53) | 0.132 |
| History of Stroke/TIA | 1.04 (0.82–1.32) | 0.734 |
| Chronic kidney disease | 1.07 (0.88–1.28) | 0.503 |
| Active cancer | 0.92 (0.66–1.27) | 0.611 |
| Coronary artery disease | 1.28 (1.05–1.55) | 0.012 |
| Diabetes mellitus | 1.18 (0.98–1.42) | 0.082 |
| Chronic lung disease | 1.28 (1.03–1.59) | 0.025 |
| Reduced functional capacity | 1.66 (1.31–2.12) | <0.001 |

Calculation with subdistributional hazard model. Abbreviations: Hb – haemoglobin, TIA – transient ischaemic attack.

**Appendix 12: Sensitivity analysis for A Cumulative incidence of all-cause mortality and B Cumulative incidence of MACE**

**
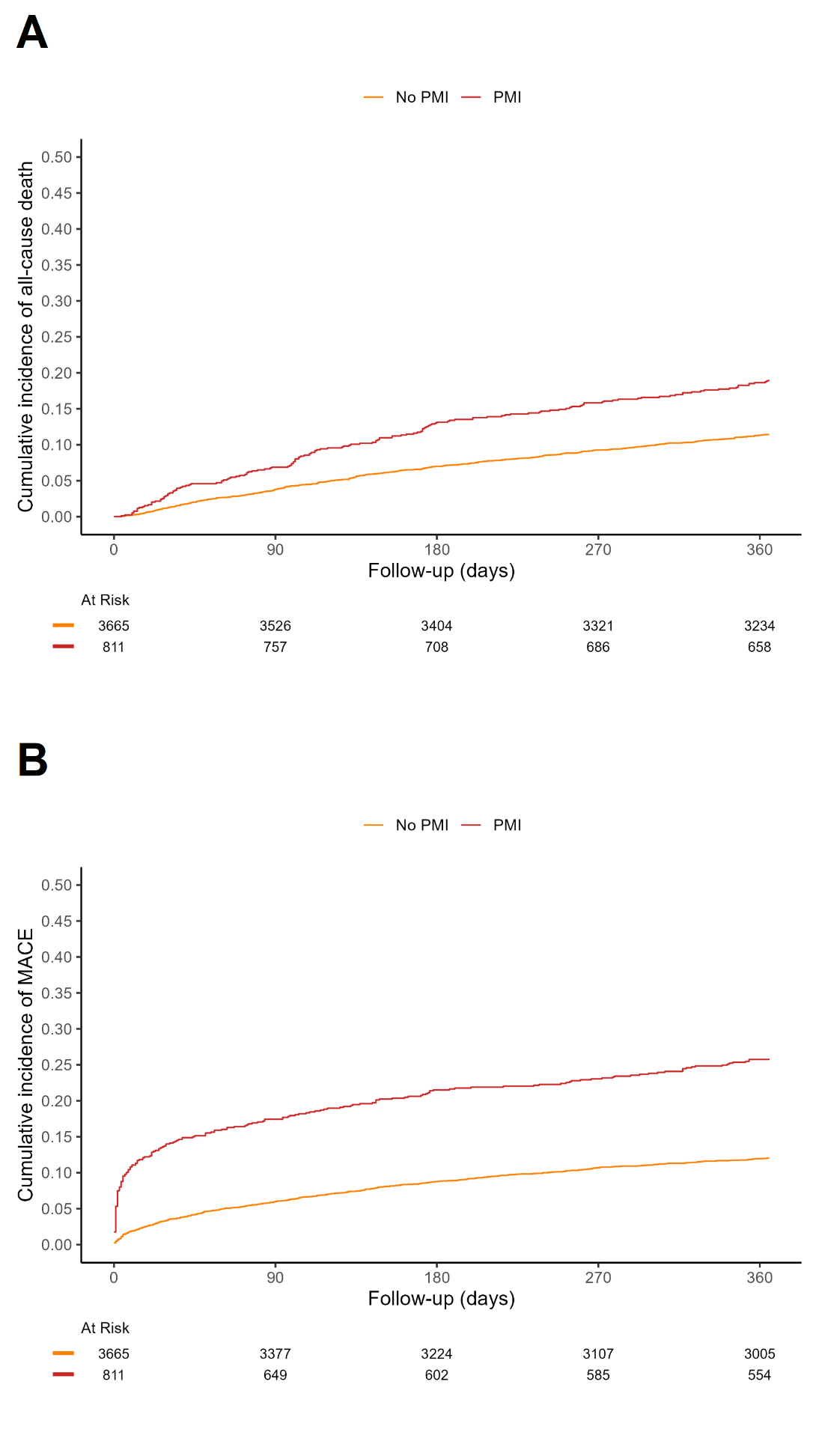
**

For sensitivity analysis, the cohort was filtered for patients alive after hospital discharge. A total of 101 patients were excluded from analysis due to missing follow-up data.

**Appendix 13: Sensitivity analysis for A Cause-specific hazard ratio of all-cause mortality and B Subdistribution hazard ratio of MACE in geriatric profile patients independent of age**

**
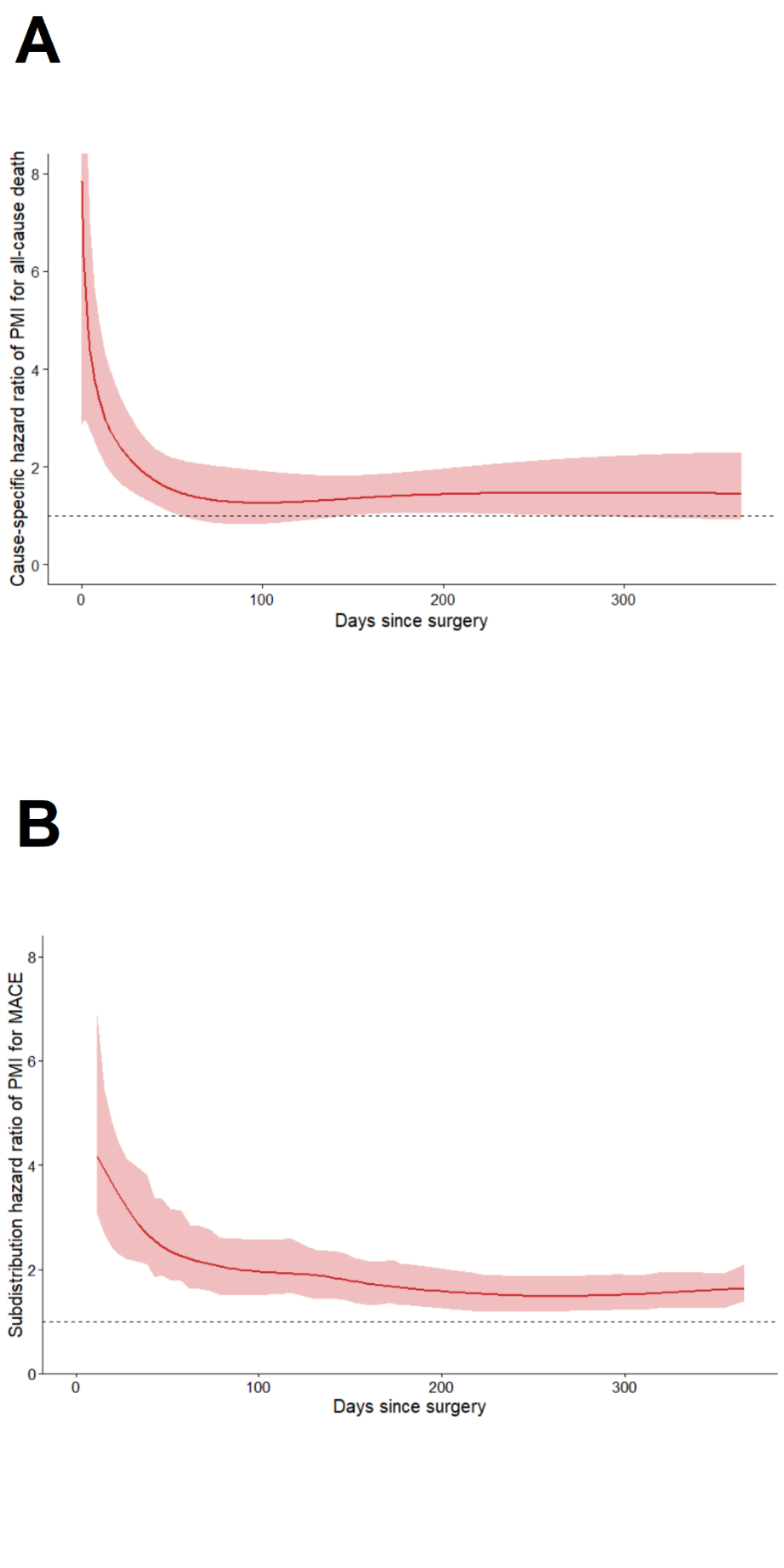
**

For sensitivity analysis, the geriatric status patients were defined independent of age. Geriatric status was defined as having ≥3 comorbidities and reduced functional capacity (<4 metabolic equivalent tasks). N = 2080.

**Appendix 14: Sensitivity analyses for A Cause-specific hazard ratio of all-cause mortality and B Subdistribution hazard ratio of MACE in age cut-offs regardless of comorbidities at I) Cut-off at 60 years, II) Cut-off at 70 years, III) Cut-off at 80 years**

1. **
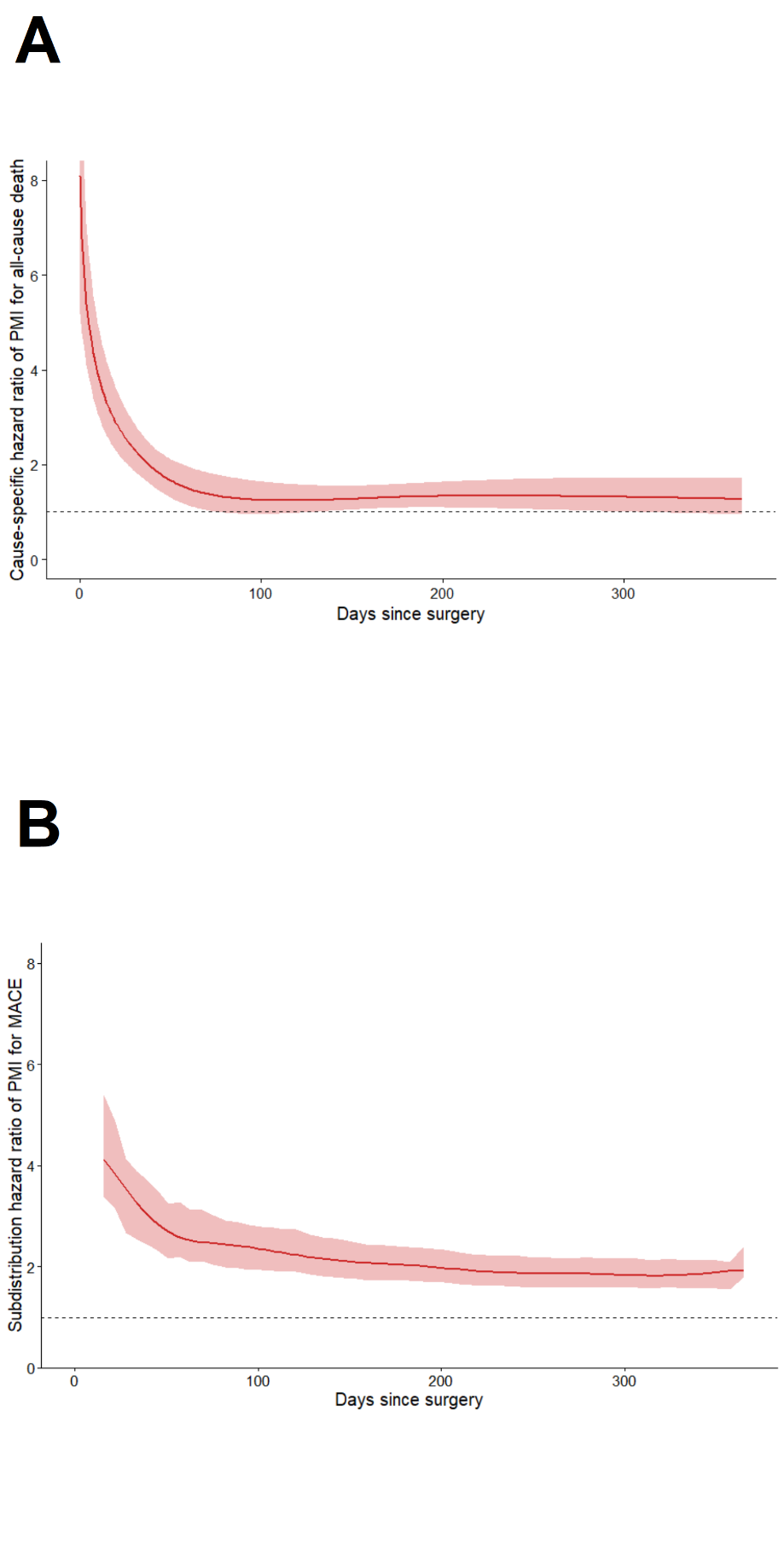
**Cut-off at 60 years (n = 10924)
2. Cut-off at 70 years (n = 7814)

**
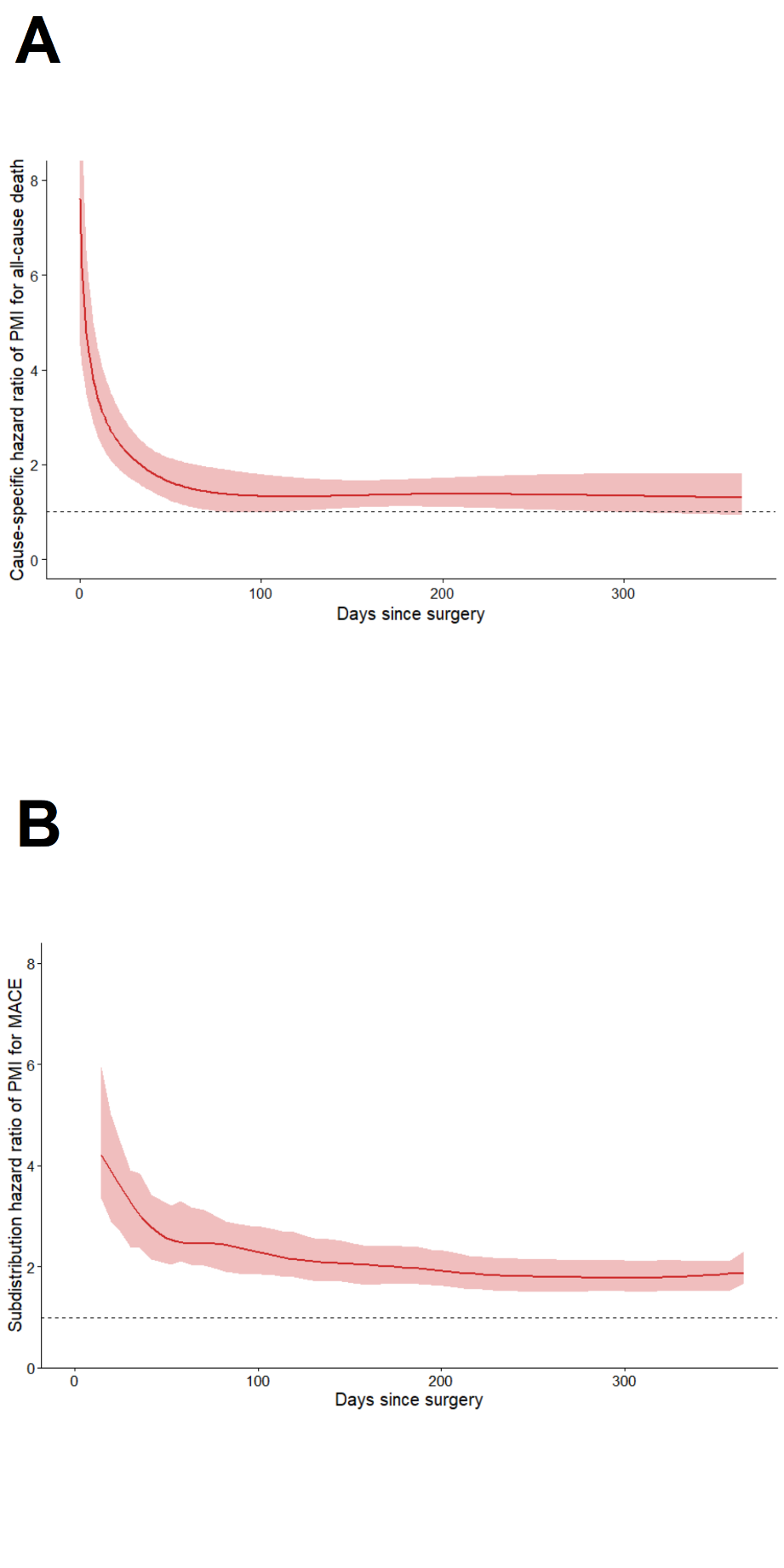
**

1.
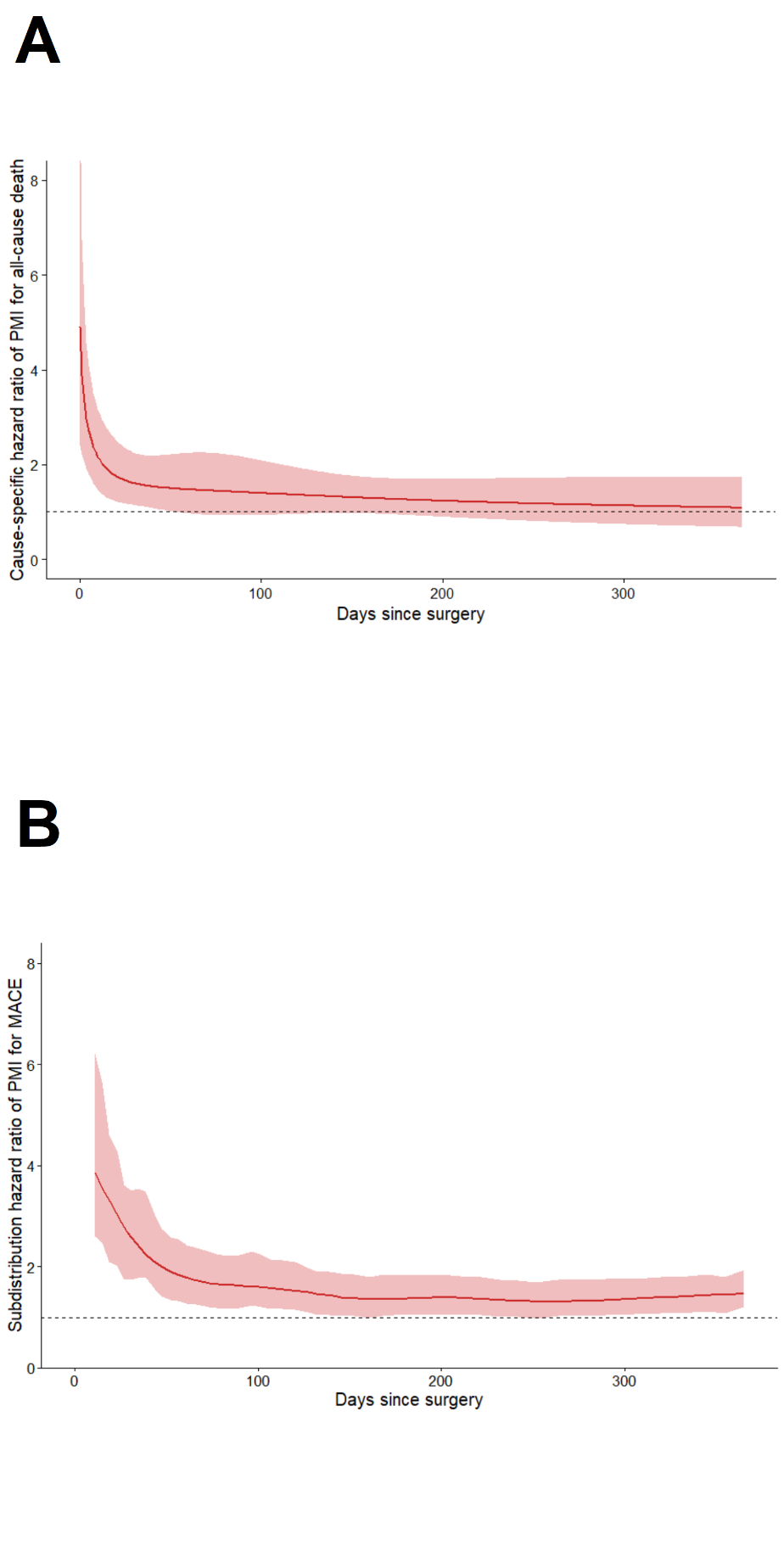
Cut-off at 80 years (n = 2641)

**Appendix 15: Results of exploratory survey of board-certified geriatricians**

**
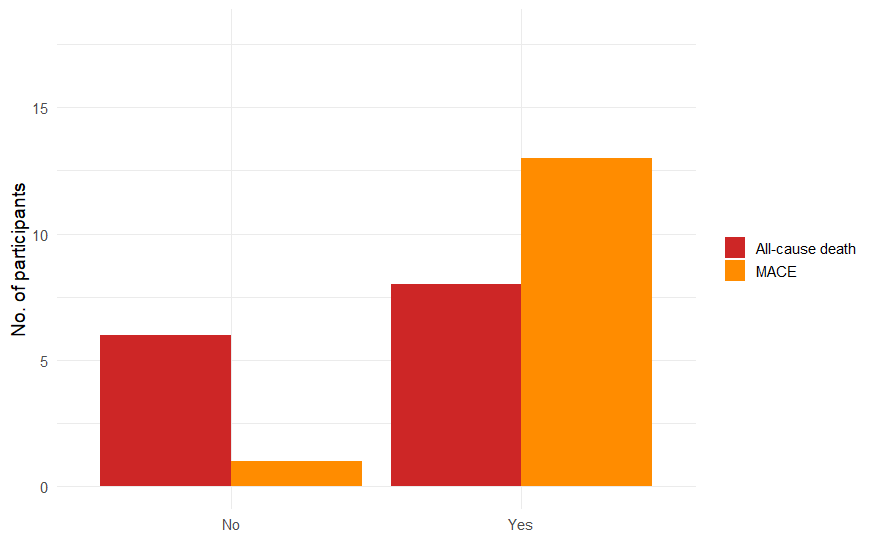
**

Results of the exploratory survey: Among the 14 board-certified geriatricians completing the survey, 6/14 (43%) did not consider PMI to be an independent predictor of all-cause mortality at 1 year. In contrast, nearly all 13/14 (93%) assumed that PMI was an independent predictor of MACE at 1 year.

**References**

1. von Elm E, Altman DG, Egger M *et al.* The Strengthening the Reporting of Observational Studies in Epidemiology (STROBE) statement: guidelines for reporting observational studies. *J Clin Epidemiol* 2008;**61**:344–9.

2. Sazgary L, Puelacher C, Lurati Buse G *et al.* Incidence of major adverse cardiac events following non-cardiac surgery. *Eur Hear J Acute Cardiovasc Care* 2021;**10**:550–8.

3. Gueckel J, Puelacher C, Glarner N *et al.* Patient- and procedure-related factors in the pathophysiology of perioperative myocardial infarction/injury. *Int J Cardiol* 2022;**353**:15–21.

4. Lurati Buse GAL, Puelacher C, Menosi Gualandro D *et al.* Association between self-reported functional capacity and major adverse cardiac events in patients at elevated risk undergoing noncardiac surgery: a prospective diagnostic cohort study. *Br J Anaesth* 2021;**126**:102–10.

5. Lurati Buse GAL, Puelacher C, Gualandro DM *et al.* Adherence to the European Society of Cardiology/European Society of Anaesthesiology recommendations on preoperative cardiac testing and association with positive results and cardiac events: a cohort study. *Br J Anaesth* 2021;**127**:376–85.

6. Puelacher C, Buse GL, Seeberger D *et al.* Perioperative myocardial injury after noncardiac surgery incidence, mortality, and characterization. *Circulation* 2018;**137**:1221–32.

7. Puelacher C, Gualandro DM, Lurati Buse G *et al.* Etiology of Peri-Operative Myocardial Infarction/Injury After Noncardiac Surgery and Associated Outcome. *J Am Coll Cardiol* 2020;**76**:1910–2.

8. Puelacher C, Gualandro DM, Glarner N *et al.* Long-Term outcomes of perioperative myocardial infarction/injury after non-cardiac surgery. *Eur Heart J* 2023;**44**:1690–701.

9. Mayet WJ. Definition of geriatric patients, relevance for gastroenterology and the geriatric assessment. *Gastroenterologe* 2021;**16**:317–23.

10. Grund S, Gordon AL, van Balen R *et al.* European consensus on core principles and future priorities for geriatric rehabilitation: consensus statement. *Eur Geriatr Med* 2020;**11**:233–8.

11. Jacobs JM, Cohen A. Changing Profile of Health and Function from Age 70 to 85 Years. 2012:313–21.

12. Stuck AK, Schilling N, Bertschi D *et al.* Predictive Abilities of the Frailty Phenotype and the Swiss Frailty Network and Repository Frailty Index for Non-Home Discharge and Functional Decline in Hospitalized Geriatric Patients. *J Frailty Aging* 2022;**11**:387–92.

13. Barni S, Luciani A, Tralongo P *et al.* Geriatric Oncology in Italy . Where we are . A CIPOMO ( Italian College of Primary Hospital Medical Oncologists ) -Gioger ( Italian Group of Geriatric Oncology ) Survey. *Clin Oncol* 2026;**49**:103965.

14. Deutsche Schmerzgesellschaft e.V. DG für G e. V and US e. V (eds. . S3-Leitlinie Schmerzmanagement bei geriatrischen Patient:innen in allen Versorgungssettings (GeriPAIN): Kurzfassung. *AWMF-Register-Nr 145/005, 2025*:7.

15. Collard RM, Boter H, Schoevers RA *et al.* Prevalence of Frailty in Community-Dwelling Older Persons : A Systematic Review. 2012:1487–92.

16. Pitter JG, Zemplényi A, Babarczy B *et al.* Frailty prevalence in 42 European countries by age and gender : development of the SHARE Frailty Atlas for Europe. *GeroScience* 2024;**46**:1807–24.

17. Caoimh RO, Sezgin D, Donovan MRO *et al.* Prevalence of frailty in 62 countries across the world : a systematic review and meta-analysis of population-level studies. 2021:96–104.

18. GBD 2019 Ageing Collaborators. Global, regional, and national burden of diseases and injuries for adults 70 years and older: systematic analysis for the Global Burden of Disease 2019 Study. *BMJ* 2022;**376**:e068208.

19. Thygesen K, Alpert JS, Jaffe AS *et al.* Fourth universal definition of myocardial infarction (2018). *Eur Heart J* 2019;**40**:237–69.

20. Halvorsen S, Mehilli J, Cassese S *et al.* 2022 ESC Guidelines on cardiovascular assessment and management of patients undergoing non-cardiac surgery. *Eur Heart J* 2022;**43**:3826–924.

21. Glarner N, Puelacher C, Gualandro DM *et al.* Association of preoperative beta-blocker use and cardiac complications after major noncardiac surgery: a prospective cohort study. *Br J Anaesth* 2024;**132**:1194–203.

22. Schmoor C, Sauerbrei W, Schumacher M. Sample size considerations for the evaluation of prognostic factors in survival analysis. *Stat Med* 2000;**19**:441–52.

23. Devereaux PJ, Biccard BM, Sigamani A *et al.* Association of postoperative high-sensitivity troponin levels with myocardial injury and 30-day mortality among patients undergoing noncardiac surgery. *JAMA - J Am Med Assoc* 2017;**317**:1642–51.

24. Pate A, Riley RD, Collins GS *et al.* Minimum sample size for developing a multivariable prediction model using multinomial logistic regression. 2023, DOI: 10.1177/09622802231151220.
